# Understanding p300-transcription factor interactions using sequence variation and hybridization†

**DOI:** 10.1039/d2cb00026a

**Published:** 2022-04-11

**Authors:** Fruzsina Hóbor, Zsófia Hegedüs, Amaurys Avila Ibarra, Vencel L. Petrovicz, Gail J. Bartlett, Richard B. Sessions, Andrew J. Wilson, Thomas A. Edwards

**Affiliations:** Astbury Centre for Structural Molecular Biology, University of Leeds, Woodhouse Lane Leeds LS2 9JT UK a.j.wilson@leeds.ac.uk t.a.edwards@leeds.ac.uk; School of Molecular and Cellular Biology, University of Leeds Woodhouse Lane Leeds LS2 9JT UK; Department of Medical Chemistry, University of Szeged Dóm tér 8 H-6720 Szeged Hungary hegedus.zsofia@med.u-szeged.hu; School of Biochemistry, University of Bristol, Medical Sciences Building, University Walk Bristol BS8 1TD UK; BrisSynBio, University of Bristol, Life Sciences Building Tyndall Avenue Bristol BS8 1TQ UK; School of Chemistry, University of Bristol Cantock's Close Bristol BS8 1TS UK; School of Chemistry, University of Leeds Woodhouse Lane Leeds LS2 9JT UK

## Abstract

The hypoxic response is central to cell function and plays a significant role in the growth and survival of solid tumours. HIF-1 regulates the hypoxic response by activating over 100 genes responsible for adaptation to hypoxia, making it a potential target for anticancer drug discovery. Although there is significant structural and mechanistic understanding of the interaction between HIF-1α and p300 alongside negative regulators of HIF-1α such as CITED2, there remains a need to further understand the sequence determinants of binding. In this work we use a combination of protein expression, chemical synthesis, fluorescence anisotropy and isothermal titration calorimetry for HIF-1α sequence variants and a HIF-1α-CITED hybrid sequence which we term CITIF. We show the HIF-1α sequence is highly tolerant to sequence variation through reduced enthalpic and less unfavourable entropic contributions, These data imply backbone as opposed to side chain interactions and ligand folding control the binding interaction and that sequence variations are tolerated as a result of adopting a more disordered bound interaction or “fuzzy” complex.

## Abstract 2

The interaction of p300 with HIF-1α is highly tolerant to sequence variations accompanied by enthalpy–entropy compensation and can be effectively inhibited by its negative regulator CITED2 with a sequence dependent competition mechanism.

## Graphical abstract

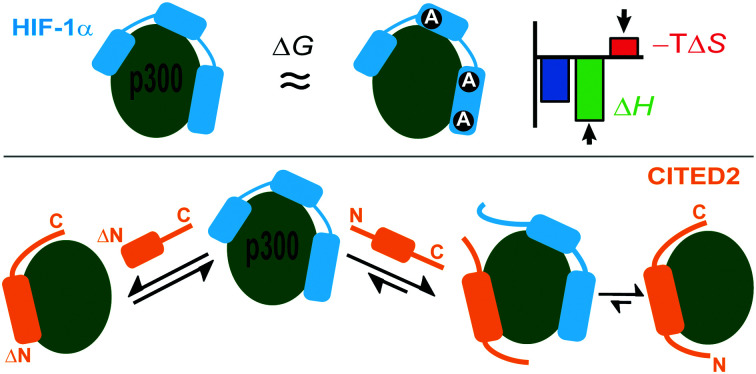

## Introduction

The hypoxic response is crucial to cell survival; it needs to both rapidly adapt to subtle variations in, and fluctuating, oxygen levels, and, allow recovery from hypoxia.^1–3^ As low oxygen level is a universal hallmark of solid tumours, the ability to adapt to hypoxia is essential for their growth and survival.^4^ The hypoxic response is mediated by transcriptional activation of genes that facilitate either short term (*e.g.* increased vascular permeability, glucose transport) or long term adaptive mechanisms (such as angiogenesis);^5–7^ these processes are largely mediated by the transcription factor Hypoxia Inducible factor (HIF) 1.^5–7^ HIF-1 is responsible for the activation of over 100 genes that play essential roles in the hypoxic response and thus plays a role in tumour growth and survival, making it a potential target for anticancer drug discovery.^8–12^ Indeed, a number of approaches to target protein–protein interactions of HIF-1 have been explored.^11,13–24^ HIF-1 is a heterodimer, consisting of two subunits, the constitutively expressed HIF-1β and the oxygen sensitive HIF-1α.^3^ Under normoxic conditions, HIF-1α undergoes hydroxylation leading to interaction with the E3 Ligase pVHL and degradation, whereas under hypoxic conditions this is suppressed resulting in accumulation and translocation of HIF-1α to the nucleus where it forms a heterodimer with HIF-1β and recruits transcriptional co-activators, such as p300.^8,25–30^ The multidomain protein p300 and its paralogue CREB binding protein (CBP) are very similar in structure; they comprise a number of domains including the nuclear interaction domain (Nu), the CREB and MYB interaction domain (KIX), cysteine/histidine regions (CH/TAZ), a histone acetyltransferase domain (HAT) and a bromodomain (Br).^31,32^ The CH1 domain (which differs by only a few amino acids between p300 and CREB^33,34^) interacts with the carboxy terminal transactivation domain (C-TAD) of HIF-1α. The CH1 domain has been shown to interact with a number of transcription factors including HIF-1α,^28,33^ CREB-binding protein/p300-interacting transactivator with ED-rich tail (CITED2),^35,36^ p53,^37^ NF-kB p65 subunit RelA,^38^ and, signal transducer and activator of transcription 2 (STAT2)^39^ through a range of recognition modes.^40^ Of particular interest, CITED2, is a negative feedback regulator that reduces HIF-1 transcriptional activity by competing for p300/CBP.^41–45^ HIF-1α and CITED2 have been reported to operate *via* a hypersensitive regulatory switch that exploits the properties of intrinsic disorder, similar p300/CBP binding affinities and a common LP(Q/E)L sequence mechanistically essential for binding, flanked by helical regions. CITED2 has been reported to displace HIF-1α from the surface of p300/CBP *via* transient ternary complex formation with both p300/CBP and HIF-1α followed by a subsequent shift in conformation resulting in a kinetic lock and prevention of the reverse process (*i.e.* displacement of CITED2 by HIF-1α).^46,47^ This provides a rationale as to why HIF-1α transcriptional activity is sensitive to moderate CITED concentrations^41^ allowing effective negative feedback.

**Fig. 1:**
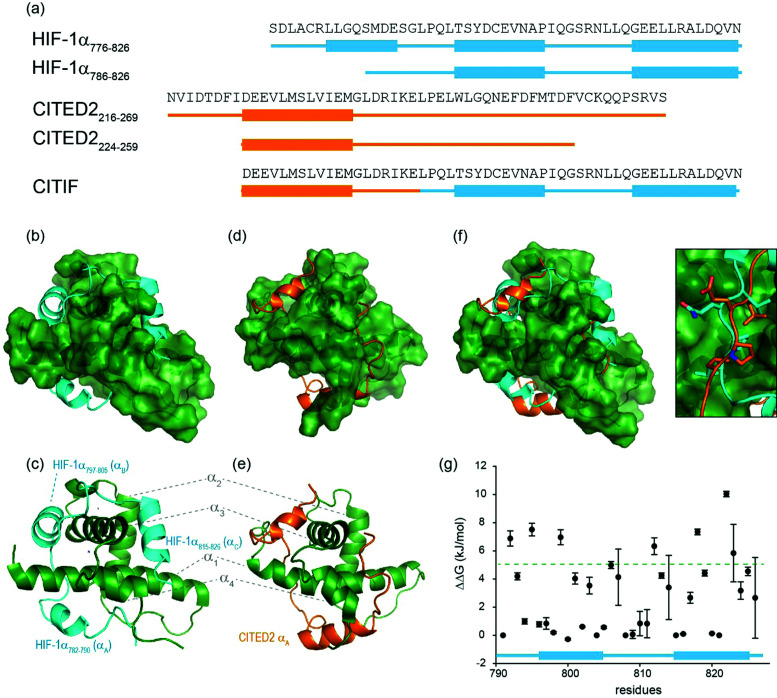
Sequences and structures of the p300 transcription factor complexes investigated in this work and binding free energy predictions on sequence determinants. (a) Sequence variants of HIF-1α and CITED2, helical regions are indicated by rectangles under the sequences. (b) Lowest energy structure from an NMR derived ensemble of the HIF-1α C-TAD (cyan fold) and CBP CH1 domain (green surface) interaction (PDB ID: 1L8C); (c) same structure with HIF-1α C-TAD (cyan fold) and CBP(p300) CH1 domain (green fold); key regions are annotated for both HIF-1α and p300 with corresponding nomenclature used by Appling *et al.*,^47^ for clarity; (d) lowest energy structure from an NMR derived ensemble of the CITED2 (orange fold) and CBP(p300) CH1 domain (green surface) interaction (PDB ID: 1P4Q); (e) same structure with CITED2 (orange fold) and CBP(p300) CH1 domain (green fold); key regions are annotated for both CITED2 and p300 with corresponding nomenclature used by Appling *et al.*,^47^ for clarity; (f) overlay of the HIF-1α C-TAD (cyan fold) and CITED2 (orange fold) interactions with CBP(p300) CH1 domain (inset highlights the region where the conserved LPE(Q)L residues interact); (g) results of hot-residue prediction using *in silico* alanine scanning (BudeAlaScan, 20 lowest energy structures from the NMR ensemble used in the prediction, circles denote average predicted ΔΔ*G*, error bars the standard deviation).

HIF-1α interacts with p300/CBP *via* its C-TAD. The solution structure of HIF-1α C-TAD in complex with p300/CBP was previously determined by NMR.^28,33^ The CH1 domain of p300/CBP forms a rigid globular structure consisting of four α-helices (referred to here as α_1–4_), stabilised and constrained by three Zn atoms. The isolated C-TAD domain of HIF-1α is disordered in the absence of its binding partner. When bound to p300/CBP the HIF-1α C-TAD consists of three distinct α-helical regions and wraps around the p300/CBP CH1 domain^28^ (Fig. 1b and c, note in structure PDB ID: 1L3E^33^ the N-terminal region does not adopt a helical conformation). Several studies provide contradictory conclusions as to the importance of various regions and residues on HIF-1α C-TAD for p300/CBP.^17,19,48,49^ Mutational studies proposed key binding residues of HIF-1α;^48^ the N-terminal helix (HIF-1α_782–790_, also referred to as HIF-1α α_A_) has been shown to be less important for p300/CBP binding whilst the central and C-terminal helices (HIF-1α_797–805_ and HIF-1α_815–826_, also referred to as HIF-1α α_B and_ HIF-1α α_C_ respectively) of the HIF-1α C-TAD have been shown to be required for p300 recognition.^50^ HIF-1α_797–805_ bears two residues, Cys800 and Asn803, which can undergo post-translational modifications that modulate binding,^15,49,51,52^ and HIF-1α_815-826_ helix residues Leu818, Leu822 and Val825 are also considered important for binding.^48^ Additional HIF-1α_815–826_ helix residues that have been suggested to be important for recognition, include Asp823 and Gln824.^17,19^ The potency of sequences derived from HIF-1α C-TAD (HIF-1α_776–826_, HIF-1α_786–826_ HIF-1α_788–822_ HIF-1α_776–813_) binding to p300/CBP were compared using fluorescence polarization.^49^ From this experiment it was concluded that the C-terminus of HIF-1α C-TAD is important for binding, in agreement with the mutagenesis studies.^33,48^ Moreover, p300 sequence variants within the region that binds HIF-1α_815–826_ highlight its importance: whilst His349Ala and Leu376Met p300 variants showed minimal difference in HIF-1α affinity, a significant drop in potency was observed for the Ile400Met p300 variant;^50^ all these variants are found within the HIF-1α_815–826_ binding region with Ile400 closest to HIF-1α_815–826_. Site-directed mutagenesis in combination with kinetics measurements have been used to study the transition state for binding p300/CBP and the HIF-1α C-TAD: 17 HIF-1α C-TAD sequence variants were generated and binding assessed. *Φ*-Value binding analysis suggested that native hydrophobic binding interactions do not form at the transition state.^53^ HIF-1α Asn-803 hydroxylation was also shown to have a minimal destabilization effect. These data suggest the rate-limiting transition state is “disordered-like”, with subsequent co-operative formation of native binding contacts and replicates results observed for other p300/CBP CH1 interactions.^54^

HIF-1α (residues 776–826) and CITED2 (residues 216–269) recognize partially overlapping binding sites on p300/CBP (Fig. 1d–f). The helices of HIF-1α and CITED2 and their conserved LP(Q/E)L motifs bind to the same surfaces of the p300/CBP CH1 domain. The region of CITED2 that is C-terminal to the LPEL motif binds in an extended conformation in the same site as the HIF-1α_797–805_ helix.^35,36^ Despite this significant structural and mechanistic understanding of transcription factor p300/CBP interactions, there is a need to further understand the determinants of binding at a sequence level. Motivated by our recent studies on the effects of the HIF-1α truncation on the HIF-1α/p300 interaction,^21,50^ identification of peptide and non-antibody binding proteins through selection methods,^50^ and, development of designed HIF-1α/p300 inhibitors^18,21,23,55^ we sought to understand those determinants. We used a combination of protein expression, chemical synthesis, fluorescence anisotropy and isothermal titration calorimetry to probe the binding of HIF-1α sequence variants, CITED2 and a HIF-1α-CITED2 hybrid sequence (which we term CITIF; Fig. 1a) to the p300 CH1 domain (residues 330–420, hereinafter referred to as p300). Our results point to an interaction that is remarkably tolerant to sequence variation, despite a high degree of sequence conservation across species.^28^ The parent interaction is enthalpically very favourable and entropically unfavourable; it seems to tolerate sequence variation through reduced enthalpic and less unfavourable entropic contributions, features which support a hypothesis whereby interactions between ligand (HIF-1α) and protein (p300) exploit a combination of non-covalent contacts between the HIF-1α backbone (as opposed to side-chains) and the surface of well folded p300 CH1 domain, along with HIF-1α folding, driven by transient side-chain contacts and long range electrostatic interactions to derive binding free energy. Adopting a more disordered bound interaction or “fuzzy” complex is consistent with the observed changes in thermodynamic signature and might account for the broadly tolerated sequences.

## Results and discussion

### HIF-1α single sequence variations have little effect on p300 binding affinity

We previously developed BudeAlaScan as a predictive tool to identify hot residues and experimentally validated it for α-helix and β-strand mediated interactions.^56,57^ In those cases the interaction was localized within a single helix or strand in at least one of the interacting partners. The extended nature of the HIF-1α/p300 interaction afforded an opportunity to test the capabilities of *in silico* alanine scanning where affinity may be dispersed across a larger number of amino acid residues (for comparison, the NOXA/MCL-1 interaction has MCL-1 binding affinity *K*_D_ ∼ 100nM with 19 residues in NOXA as opposed to HIF-1α with similar *K*_D_ but 42 residues). BudeAlaScan can predict the difference in binding upon introducing single or multiple alanine variations in one of the interacting partners when compared to the binding energy of the wild-type protein (ΔΔ*G* = Δ*G*_WT_ − Δ*G*_variant_); in this case for HIF-1α using the HIF-1α/CBP NMR derived ensemble (PDB ID: 1L8C). This analysis (Fig. 1g) predicted key determinants of the HIF-1α binding to be dispersed across the whole sequence with several residues in both HIF-1α_797–805_ and HIF-1α_815–826_ showing ΔΔ*G* > 4.2 kJ mol^−1^ (the threshold for a hot residue).^57–59^ A number of these *e.g.* L792 and L822 show ΔΔ*G* ≫ 4.2 kJ mol^−1^ with small standard deviation implying those positions are indeed important for p300 binding while other residues with smaller values and greater standard deviation, like D823 were less clear cut. ROBETTA^60^ provided similar data (see ESI,† Fig. S1).

**Fig. 2:**
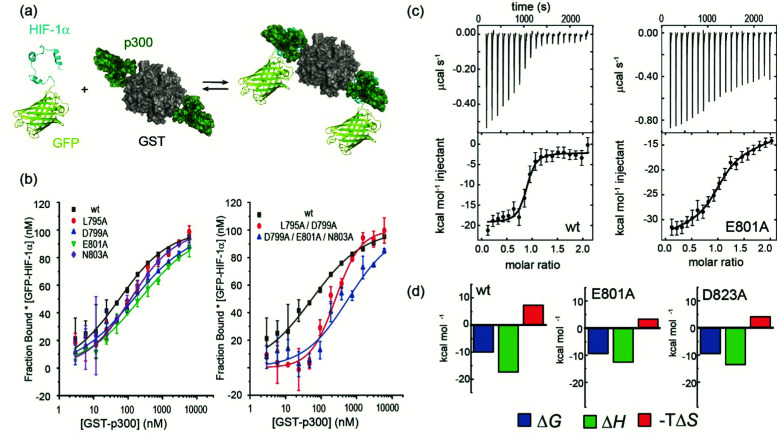
Biophysical analyses on the effects of HIF-1α sequence variant p300 binding affinity. (a) schematic depicting the equilibrium for interaction of GFP-HIF-1α_776–826_ variants and GST-p300 as studied by fluorescence anisotropy; (b) representative fluorescence anisotropy titration data for sAV and mAV GFP-HIF-1α_776–826_ peptides interacting with GST-p300 (25 mM Tris–HCl, 150 mM NaCl, 1 mM DTT, pH 7.4), error bars represent standard deviation of technical triplicates; (c) raw ITC data (upper) and fitted thermogram (lower) for the interaction of GFP-HIF-1α_776–826_ peptides with p300 (37 °C in 25 mM Tris–HCl, 150 mM NaCl, 1 mM DTT, pH 7.4) using 10 μM p300 in the cell and 100 μM GFP-HIF-1α_776–826_ variant in the syringe; error bars represent estimated integration errors (d) thermodynamic signatures for each interaction.

To experimentally compare the predictions, we carried out an *in vitro* biophysical study of several HIF-1α sequence variants. We assessed predicted hot residues and their interactions with p300 using the NMR structure to visualize the structural basis behind the predictions (See ESI,† Fig. S2). These analyses helped refine a first series of alanine variants to prepare. We did not consider HIF-1α_782–790_ variants given prior studies which had established little overall effect from the presence/absence of these 8–10 residues.^50^ HIF-1α_776–826_ sequence variants were recombinantly prepared based on the predictions to test their binding to the recombinantly prepared p300 (Fig. 2). Given the length of the peptide (42 residues), this was considered advantageous as it obviates the need to chemically synthesise, label and purify multiple variants. As the C-TAD domain is unstructured in isolation it was recombinantly expressed as a fusion protein with GFP. The green-fluorescent protein (GFP) tag was used for fluorescence anisotropy (FA) experiments to determine the binding affinity of HIF-1α_776–826_ C-TAD variants to p300. As the CH1 domain of p300 is a small domain of 11 kDa it was recombinantly expressed as a fusion protein with GST to increase its size and thus the signal to noise in the FA experiments. We established an assay where the interaction between GFP-tagged wt HIF-1α_776–826_ and GST-tagged p300 was monitored by FA, using the fluorescence of GFP. GST dimerises with single digit nM *K*_d_^61^ (and in our hands exists as a dimer in size exclusion chromatography analyses, see ESI,† Fig. S15 for protein characterization data), thus two molecules of GFP-HIF-1α could bind to the GST-p300 dimer (Fig. 2a). We make the assumption that each p300 binding site behaves independently and analyse the binding isotherm using a 1 : 1 model. This is a reasonable assumption given (i) that binding affinity between HIF-1α and p300 appears to be unaffected by GST, (ii) the theoretical fluorescence anisotropy difference between the 1 : 2 and 2 : 2 complexes is negligible. A control experiment was performed using GFP-HIF-1α_776–826_ and p300 with the GST tag cleaved; although the change in anisotropy signal was lower (consistent with the lower mass of the complex in the absence of GST) the determined *K*_D_ was comparable between the two experiments (Fig. S3, ESI†). Similarly, ITC experiments for the binding of GFP-HIF-1α_776–826_ and HIF-1α_776–826_ to p300 were comparable (see later).

After establishing the assay, selected single alanine HIF-1α C-TAD variants (sAVs) were prepared and their binding affinity to p300 was tested. Results for these experiments are shown in Fig. 2b, Table 1 and Fig. S4 (ESI†). Our data show there is a limited impact upon the binding to p300 for single alanine variations introduced into HIF-1α_797–805_, HIF-1α_815–826_ or the LPQL sequence that shares homology with CITED2 (≤4 fold maximal difference). This contrasts with the work of Lindström who identified L792A, L795A and L818A as hot residues (alongside L812A and L813A, which were not considered here), although these were derived from *Φ*-value binding analysis using tryptophan fluorescence and may reflect transition state effects upon binding.

**Table 1:** Dissociation constants (with standard error of the fitted value) for GFP-HIF-1α_776–826_ C-TAD single alanine mutant variants binding to GST-p300

HIF-1α SAV variant	*K*_D_ (nM)^a^	HIF-1α MAV variant	*K*_D_ (nM)^a^
wt	55 ± 27	L795A D799A	255 ± 44
L795A	118 ± 19	D799A E801A N803A	590 ± 302
S797A	37 ± 16	L818A L822A	126 ± 33
D799A	114 ± 53	L818A L822A D823A	247 ± 75
E801A	227 ± 78	L818A L822A V825A	54 ± 33
N803A	120 ± 17	E801A L822A	224 ± 18
E817A	128 ± 11	L795A D799A L822A	174 ± 45
L818A	237 ± 40	L795A D799A L818A L822A V825A	153 ± 19
L822A	109 ± 66		
D823A	192 ± 17		
Q824A	31 ± 8		
V825A	198 ± 37		

a Conditions as in Fig. 2b.

We carried out isothermal titration calorimetry (ITC) measurements for several variants to verify the results of the fluorescence anisotropy measurements (Fig. 2c and ESI,† Fig. S5). The interaction of HIF-1α_776–826_ with p300 is characterized by a large favourable enthalpy of interaction and opposing unfavourable entropy of interaction. The dissociation constant was similar for all the tested variants and the thermodynamic signature shifted toward less favourable enthalpic contributions compensated by more favourable entropy (Fig. 2c and d, Table 2.) The removal of a transient charge reinforced interaction (E801A and D823A) may increase the local flexibility of the structure resulting in the observed, less unfavourable entropy. This implies that HIF-1α can adjust its interaction with p300 to achieve optimal affinity also for the variants, which emphasizes the requirement to occupy the surface through ‘fuzzy’ interactions rather than specific contacts. However, we cannot on the basis of these data, exclude a role for differential solvation of the sequence variants inducing these thermodynamic differences, it is well known that enthalpy–entropy compensation can arise from structural reorganization of solute hydrating hydrogen-bonding networks,^62–65^ and within this context it is important to acknowledge that the effects of a local perturbation (*e.g.* a side chain interaction), can be masked by global changes (*e.g.* to conformation) induced by the perturbation.^66^

**Table 2:** Thermodynamic parameters for the binding of GFP-HIF-1α_776–826_ variants to p300. Data were fitted to a fixed 1 : 1 stoichiometry, including a baseline and incompetent protein fraction fitting. 68% confidence intervals for the fitted values are shown in brackets (conditions as in Fig. 2c)

	*K*_D_ nM	Δ*H* kcal mol^−1^	Δ*S* cal mol^−1^ K^−1^
HIF-1α	86 (32–182)	−17.3 (−18.1 to −16.6)	−23.6
HIF-1α E801A	359 (284–444)	−12.2 (−12.6 to −11.9)	−9.5
HIF-1α D823A	369 (264–496)	−13.0 (−12.5 to −13.6)	−12.2
HIF-1α L792A	386 (320–459)	−13.6^a^ (−13.9, −13.2)	−14.5^a^

a These values should be treated with caution due to a small number of titration points (see Fig. S5, ESI).

### HIF-1α multiple sequence variations do not affect p300 binding affinity

**Fig. 3:**
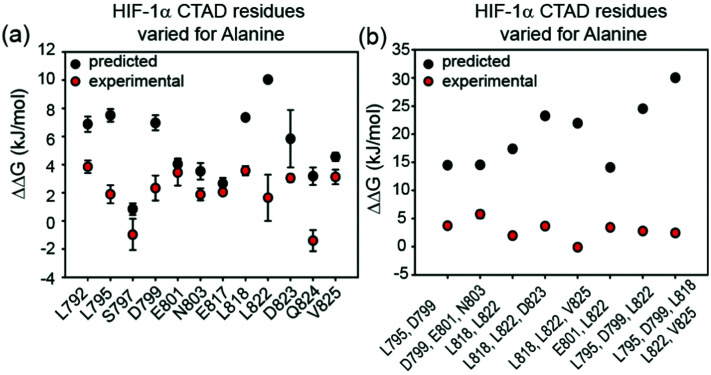
Comparison of predicted and experimental ΔΔ*G* values for: (a) single alanine variant (sAV) and (b) multiple alanine variants (mAV) of HIF-1α C-TAD for binding to p300. ΔΔ*G* values were derived from FA measurements, except for L792A for which ITC data was used.

To assess the extent to which sequence variations could confer additive effects on binding affinity, different, structurally relevant combinations (*i.e.* with the highest combined predicted ΔΔ*G* values) of alanine variations were introduced into the HIF-1α_776–826_ C-TAD and their binding affinity determined (Fig. 3, Table 1 and Fig. S4, ESI†). The experimental data for these multiple alanine variants (mAVs) clearly shows that variations of two or three predicted hot residues either in HIF-1α_797–805_ or HIF-1α_815–826_ are generally insufficient to abrogate p300 binding. Even introducing variations in two helices (*e.g.* E801A L822A) simultaneously did not increase the *K*_D_ significantly; variants generally maintained affinity to p300 although for some mAVs (*e.g.*, D799A–E801A–N803A; L795A–D799A; L818A–L822A–D823A and E801A L822A), there appears to be some loss in potency. Lower net negative charge of TADs influences long-range electrostatic interactions leading to lower association rates,^67^ which, in part may explain the decreased binding affinity of some of these mAVs. Collectively, these data further support a conclusion that the HIF-1α/p300 interface is fuzzy in nature; the plasticity in the interaction allows for signficiant sequence variation in the HIF-1α C-TAD with loss of one side chain likely to be compensated for by interactions of other side chains, possibly augmented by interactions of the backbone with the p300 surface.

### Comparison of predicted and experimental variant HIF-1α/p300 binding affinities

The experimentally determined values for sAVs and mAVs do not agree fully with the predictions (Fig. 3). It should be noted that the predictions (both using BudeAlaScan and ROBETTA) did not identify particularly large sAV ΔΔ*G* values > 8 kJ mol^−1^ and our earlier work highlighted the challenges in accurately predicting absolute values of ΔΔ*G* using fast methods which are well suited to a yes/no indicator.^56,57^ Overall, the comparison between prediction and experiment for sAVs reveals the predictions overestimate the change in affinity, although there is still a moderate effect for most predicted hot-residues. Comparison of the prediction and experiment for mAVs reveals more pronounced differences; the additive combination of sequence variations is predicted to be significant (>15 kJ mol^−1^ in many cases), yet minimal effects are observed for as many as five simultaneous sequence variations. This is consistent with the interaction becoming fuzzier upon sequence variation to compensate for loss of side-chain interactions, a property not assessed in predictive alanine scanning.

Taken together our results suggest that interaction of some of the side-chains from each helix of HIF-1α are sufficient to maintain nanomolar affinity for p300; as the three helices wrap around p300; varying one or two positions is not sufficient to disrupt the binding, implying a high degree of chelate co-operativity (observed in our earlier truncation studies)^50^ and dispersal of binding energy across the sequence. As noted above for the thermodynamic analyses, the large favourable enthalpy and unfavourable entropy of binding for the native HIF-1α/p300 interaction together with the well tolerated sequence variation and observed enthalpy–entropy compensation for variants predicted to have diminished p300 affinity points to a key role of backbone or long range electrostatic interactions and transcription factor folding to generate binding energy. Such behaviour and any potential decrease of unfavourable steric contacts would accommodate sequence variation where the variant bound complex is more disordered relative to the native bound complex. As these features are not explored using computational alanine scanning, the predicted affinity change upon mutation using a static structure can be misleading for such dynamic complexes. In general, the distribution of hot residues across the interaction interface with low predicted ΔΔ*G* values may be indicative of a more disordered structure.

### CITED2 has higher affinity than HIF-1α for p300 and exhibits a sequence dependent competition mechanism

We hypothesized that it would be possible to enhance the affinity of HIF-1α for p300 by hybridising key regions of both CITED2 and HIF-1α (see later). We first measured the affinity of the parent peptides. A particular difficulty in comparing these peptide sequences is the different length used in different studies.^28,33,35,36^ We therefore considered HIF-1α_786–826_, HIF-1α_776–826_ CITED2_224–259_ and CITED2_216–269_ for these analyses and studied binding to p300 using ITC. Initially we expressed these as GFP fusion proteins and cleaved the tag, however the peptides all contained four residues from the PreScission protease sequence (ITC data given in the ESI† Fig. S6 and Table 3).

**Table 3:** Thermodynamic parameters for the binding of HIF-1α, CITED2 and CITIF peptides to p300. Data were fitted to a fixed 1 : 1 stoichiometry, with baseline and incompetent protein fraction as adjustable parameters. 68% confidence intervals for the fitted values are shown in brackets

	Synthetic peptides^a^			Expressed peptides^b^		
	*K*_D_ (nM)	Δ*H* kcal mol^−1^	Δ*S* cal mol^−1^ K^−1^	*K*_D_ (nM)	Δ*H* kcal mol^−1^	Δ*S* cal mol^−1^ K^−1^
HIF-1α_786–826_	42.9 (38.3 – 47.6)	−25.8 (−25.6 to −26.1)	−50	88.3 (57.0–132.4)	−32.7 (−31.5 to −33.9)	−73.2
HIF-1α_776–826_	52.2 (49.2–55.8)	−22.9 (−22.8 to −23.1)	−41	82.4 (55.0–119.5)	−22.2 (−21.5 to −22.9)	−39.2
CITED_224–259_	9.1 (6.5–12.2)	−12.4 (−12.2 to −12.7)	−3.6	41.9 (28.9–59.5)	−12.4 (−12.0 to −12.7)	−6.2
CITED_216–269_	26.3 (20.8–33.1)	−13.2 (−12.9 to −13.4)	−10.0	18.8 (12.2–27.6)	−13.3 (−13 to −13.5)	−7.5
CITIF	11.3 (9.3–13.7)	−22.5 (−22.3 to −22.7)	−37.8	15.4 (10.4–21.8)	−18.3 (−17.9 to −18.7)	−23.3

a Conditions as in Fig. 4.

b Conditions as given in Fig. S6 (ESI).

Subsequently we also developed a chemical synthesis of the peptides bearing an N-terminal acetamide and C-terminal amide (see ESI†). In general, both sets of reagents gave similar data in terms of *K*_D_ – one notable exception is CITED2_224–259_ which gave a *K*_D_ four-fold lower in magnitude for the expressed peptide relative to the chemically synthesized peptide (Fig. 4 and Table 3.). It may be that the four residues (Gly-Pro-Gly-Ser) remaining from the PreScission protease cleavage or free N-terminus interfere with p300 recognition. Support for this hypothesis is strengthened by the fact that both HIF-1α sequences also have weaker affinity (although not as pronounced) in comparison to the synthetic peptides. Overall, the CITED2 peptides have slightly higher p300 affinity than the HIF-1α peptides. This differs from observations reported by Berlow *et al.* who observed identical *K*_D_s of 10 nM for HIF-1α_776–826_, 10 nM CITED2_216–269_ both labelled with Alexafluor 488/595. In this prior work, a variety of biophysical and NMR methods were used to show that despite similar potencies, CITED2 effectively displaces HIF-1α from the surface of p300 *via* transient ternary complex formation with both p300 and HIF-1α followed by a subsequent shift in conformation resulting in a kinetic lock and suppression of the reverse process (*i.e.* displacement of CITED2 by HIF-1α).^46^ Although the NMR experiments were performed at higher concentration, the fluorescent experiments used to determine affinity were performed at lower concentrations; the fluorescent labels and their positions may influence the equilibrium. The ITC data on unlabelled peptides which we report here suggest that the moderate preference for interaction of CITED2 with p300 over HIF-1α may incorporate a thermodynamic aspect and not exclusively derive from kinetic factors.

**Fig. 4:**
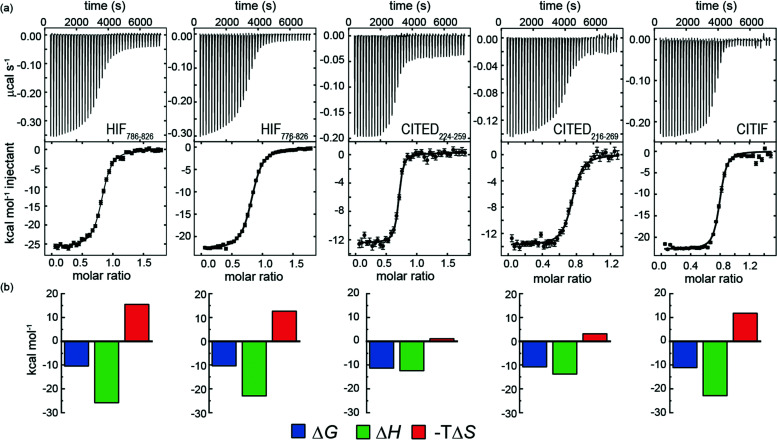
(a) Isothermal titration calorimetry data for the interaction of chemically synthesized HIF-1α, CITED2 and CITIF peptides with p300. Raw ITC (upper) data and fitted thermogram (lower) (40 mM sodium phosphate, pH 7.5 100 mM NaCl, 1 mM DTT buffer using 5 μM protein in the cell and 60 μM ligand in the syringe at 35 °C) error bars represent the estimated integration errors; (b) Thermodynamic signatures for each interaction.

**Fig. 5:**
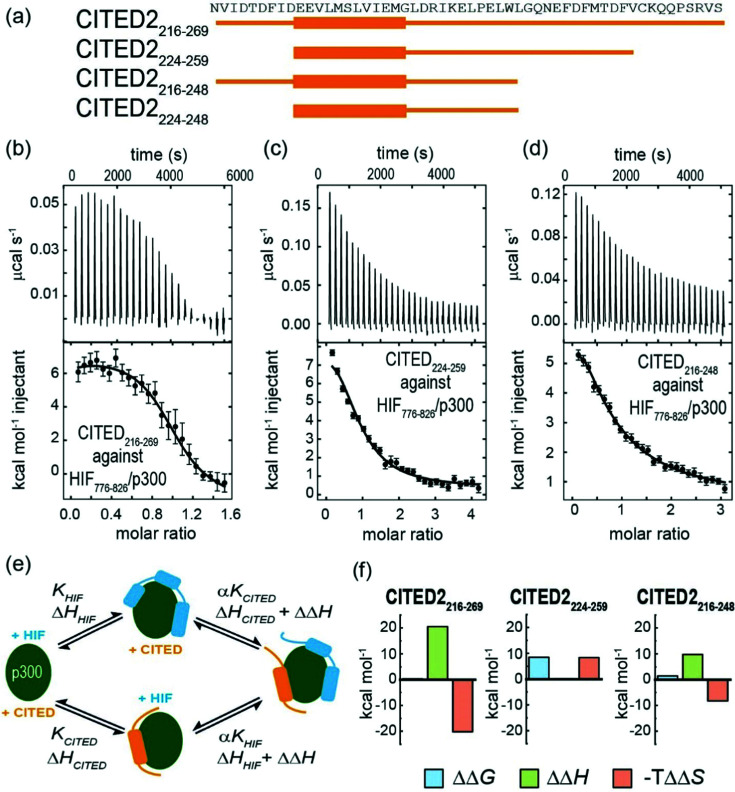
(a) CITED2 sequences used in competition experiments. Competition ITC experiments: raw ITC (upper) data and fitted thermogram (lower) for (b) CITED2_216–269_ (c) CITED2_224–259_ and (d) CITED2_216–248_ titrated against the p300/HIF-1α_776–826_ complex (40 mM sodium phosphate, pH 7.5 100 mM NaCl, 1 mM DTT buffer at 35 °C). Complexes were prepared by titrating p300 with the competitor ligand until it reached saturation, which resulted in 1.2–1.6 equivalent ligand in the cell (Table S5, ESI†) Error bars represent the estimated integration errors. (e) Arrow diagram showing the different equilibria for the CITED2-HIF-1α-p300 system (green: p300, cyan: HIF-1α, orange: CITED2), α = cooperativity constant. (f) Cooperative thermodynamic parameters extracted from the global fitting, ΔΔ*G* was derived from α (see ESI† eqn (S5)–(S9).) Fitted enthalpograms are shown in Fig. S9 (ESI†). ΔΔ*G*/ΔΔ*H* are the difference between the free energy/binding enthalpy of the ternary complex and the sum of the free energy/enthalpy measured separately for each binary complex and reflects cooperative parameters when CITED2 binds to the preformed HIF-1α/p300 complex.

To gain insight into the competition between CITED2 and HIF-1α we performed ITC experiments by titrating CITED2 sequences of different length into a preformed complex of HIF-1α_776–826_/p300, including two further truncated CITED2 versions (Fig. 5a). The data were fitted to a competition model (Fig. S17, ESI†). CITED2_216–269_ effectively displaced HIF-1α_776-826_ with an apparent *K*_D_ (*K*_D_,app) of 1.1 nM (Table 4 and Fig. 5b) which is 24-fold lower than its *K*_D_ to p300, whereas in the reverse process little binding could be detected (Fig. S7a, ESI†). This suggested that there is an underlying cooperative process that renders this sequence a more potent inhibitor than would be expected based on its binding affinity to p300 alone. This is in line with the unidirectional competition mechanism but slightly differs from the value reported by Berlow *et al.* using fluorescence anisotropy: apparent *K*_D_ for CITED2 against the p300/HIF-1α = 0.2 nM, which is 50-fold lower value than the *K*_D_ determined for the direct CITED2/p300 interaction (*K*_D_ =10 nM). The shorter CITED2_224–259_ displaced HIF-1α_776–826_ less efficiently and its apparent *K*_D_ = 12.4 nM, is similar to its binding affinity to p300 (Table 4 and Fig. 5c), which is expected for ligands that bind competitively without an allosteric contribution. The titration performed with p300/HIF-1α_786–826_ revealed similar results (Fig. S7b, c and Table S1, ESI†). Despite the fact that both CITED2 sequences contain the cooperatively acting binding motifs (the helical CITED2_224–235_ residues, LPEL motif and the aromatic/hydrophobic residues of CITED2_247–260_)^68^ that are required for allosteric function and the switch-like inhibition, our data indicate that there is a mechanistic difference between the two. Since the C-terminus of CITED2 is mainly unstructured, we hypothesize that the N-terminal CITED2_216–224_ residues may play a role in rendering the interaction unidirectional. The C-terminally truncated CITED2_216–248_ had ∼30-fold higher *K*_D_ to p300 (*K*_D_ =303 nM, Fig. S8 and Table S2, ESI†) but displayed efficient displacement of HIF-1α_776–826_ with a *K*_D,app_ = 46.9 nM only 4-fold higher compared to CITED2_224–259_ (Table 4, Fig. 5d), indicating an allosteric role of the N terminal residues. In the absence of the eight N-terminal residues (CITED2_224–248_) the affinity to p300 decreased significantly (*K*_D_ = 10 μM) (Fig. S8 and Table S2, ESI†), preventing us from performing the competition experiments but highlighting the importance of the N-terminal residues for binding.

**Table 4:** Comparison of direct binding to p300 (*K*_D_, fitted to a single binding site model) and competition against the preformed p300/HIF_776–826_ complex (*K*_D,app_, fitted to a competitive binding model depicted in Fig. S17, ESI) for the different CITED2 peptides. 68% confidence intervals of the fitted values are shown in brackets. Further thermodynamic parameters, and enthalpograms are listed in Tables S1 and S2 and Fig. S8 (ESI)

	Titration to p300 *K*_D_	Titration to HIF-1α_776–826_/p300 complex *K*_D,app_
CITED2_216–269_	26.3 nM (20.8–33.1)	1.1 nM (0.9–1.4)
CITED2_224–259_	9.1 nM (6.5–12.2)	12.2 nM (11.2–13.1)
CITED2_216–248_	303 nM (230–397)	46.9 nM (39.6–55.6)
CITED2_224–248_	10.4 μM (7.8–14.2)	n.a.

a Conditions as in Fig. 5.

To gain more insight into the mechanism by which the different CITED2 sequences displace HIF-1α from the preformed HIF-1α/p300 complex, we performed global fitting^69^ of the competition data using a model that permits ternary complex formation depicted in Fig. 5e (fitted thermograms are shown on Fig. S9 (ESI†), for detailed analysis see Fig. S18 and ESI† eqn (S5)–(S20)). The reported cooperativity constant (*α*), the derived ΔΔ*G* and ΔΔ*H* represent the additional Gibbs energy and additional contribution to the enthalpy due to cooperative interactions and indicate whether the ternary complex formation is favourable (negative ΔΔ*G*) or unfavourable (positive ΔΔ*G*).^70,71^ For CITED2_216–269_ and CITED2_216–248_ the fitted small positive ΔΔ*G* values indicated negative cooperativity, meaning that these sequences bind only moderately weaker in the presence of HIF-1α, allowing the formation of a slightly destabilized transient ternary complex with an entropy driven stabilization (Fig. 5f and Table S3, ESI†). The predicted titration curve for the reverse process (HIF-1α_776–826_ titrated to preformed CITED2_216–269_/p300) using the obtained parameters did not match with the experimental data (Fig S10, ESI†) which is in line with the model in which the equilibria are strongly shifted toward the CITED2 bound conformation due to a kinetic lock. For CITED2_224–259_ the high positive ΔΔ*G* value (Fig. 5f) indicated that the formation CITED2_224–259_/HIF-1α/p300 ternary complex is strongly unfavourable, the two ligands bind with maximum negative cooperativity (competitive ligands) and as a consequence there is no allosteric process involved in the competition mechanism. This is in line with our observations regarding the apparent *K*_D_ values discussed above and suggests that together with the other binding motifs the N-terminal residues of CITED2 are also required for transient ternary complex formation allowing the allosteric regulation and subsequent switch-like displacement of HIF-1α.

### A HIF-1α-CITED2 hybrid – CITIF – has comparable p300 binding affinity to CITED2, but exhibits intermediate enthalpic and entropic signature to those of the parent HIF-1α and CITED2 sequences

**Fig. 6:**
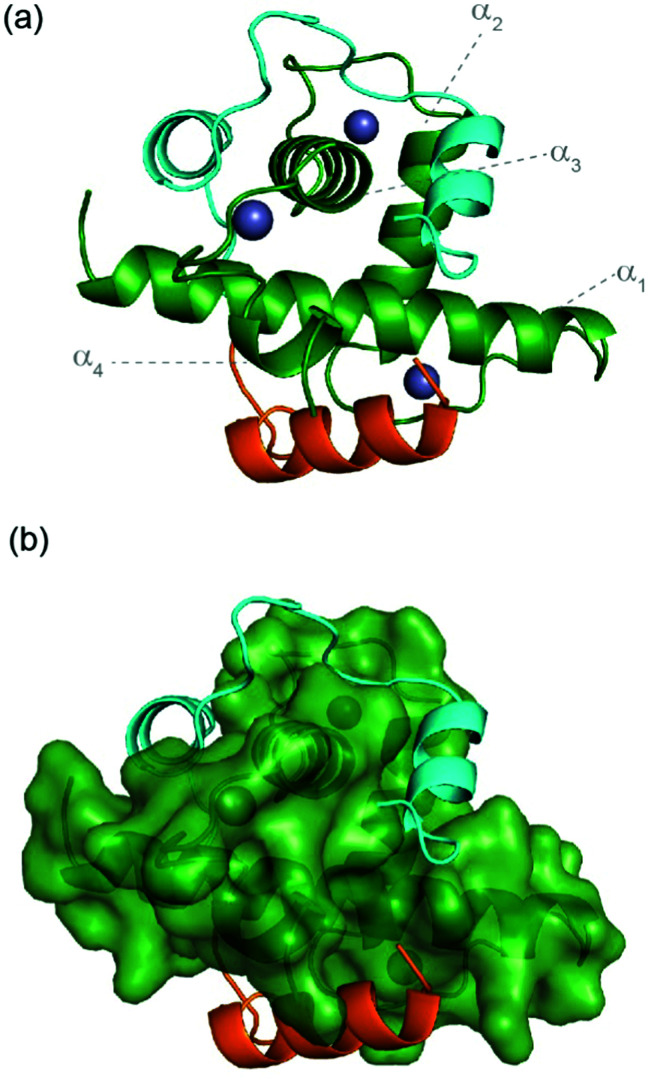
Crystal structure of p300 (green) in complex with CITIF determined at 2 Å resolution (PDB: 7QGS). Residues corresponding to CITED2_224–243_ are coloured orange, residues corresponding to HIF-1α_792–826_ are coloured cyan.

A hybrid sequence (CITIF) was designed containing an N-terminal fragment of CITED2 (224–243) and a C-terminal fragment of HIF-1α (792–826) fragment. Expressed and chemically synthesized peptides were tested with both giving a *K*_D_ slightly lower than the HIF-1α sequences and comparable to the CITED2 sequences (Table 3). A fluorescence anisotropy competition assay established that this hybrid sequence competes with HIF-1α for binding to p300, supporting the hypothesis that CITIF reproduces key binding features of both HIF-1α and CITED2 (Fig. S11, ESI†). Whilst both the HIF-1α sequences were shown to have strongly favourable p300 binding enthalpies and strongly unfavourable p300 binding entropies, in contrast, the CITED2 sequences were shown to have much less favourable p300 binding enthalpies, and much less unfavourable p300 binding entropies. The CITIF sequence exhibited p300 binding enthalpies and entropies intermediate between those observed for HIF-1α and CITED2. We obtained co-crystals of p300 in complex with CITIF and solved the structure at 2 Å resolution (Fig. 6 and Table S4, ESI†). Overall, the high B-factors and the poor electron density especially for the loop regions point to the dynamic nature of the complex, which is compensated by the tightly packed crystal lattice with an unusually low solvent content (25.7%) resulting in high resolution. The structure shows that residues corresponding to CITED2 and HIF-1α bind simultaneously, occupying their native binding sites and reproducing most of the native contacts with the protein (Fig. S13, ESI†), in line with the thermodynamic signature we observed for CITIF binding. Similarly to the CITED2-HIF-1α fusion peptide/CBP complex (PDB: 7LVS, Fig S14, ESI†) recently reported by Appling *et al.*, the N-terminal p300_345–373_ helix (α_1_) is straightened compared to the CITED2/p300 and HIF-1α/P300 binary complexes and the C terminus of CITIF (corresponding to HIF-1α_815–826_) is not fully folded, which might be due to the allosteric effects of CITED2 residues binding.

These data show that CITED2_224–243_ and HIF-1α_792–826_ sequences can bind simultaneously to p300 without interfering with one another, further supporting the formation of a ternary complex. Berlow *et al.* previously used ^1^H–^15^N NOE experiments to identify significant differences in the degree of dynamic disorder and therefore flexibility between p300 bound ^15^N HIF-1α and ^15^N CITED2.^46^ HIF-1α was shown to display a wide range of dynamics throughout its sequence with both ordered and flexible regions, notably in the LPQL motif which was shown to play a role in the binding mechanism. CITED2 on the other hand elicited more uniform behaviour consistent with a more ordered structure. Subsequently Appling *et al.* used a HIF-1α-CITED2 fusion peptide similar to the one reported here to probe further the binding mechanism; these studies revealed that the region corresponding to HIF-1α_815–826_ and the region corresponding to the CITED2_224–235_ are mutually destabilizing to one another and this negative allostery is governed by the length and orientation of the C-terminal p300 helix (α_4_).^47^ Molecular dynamics simulations also identified a HIF-1α/CITED2/p300 ternary complex in support of this model and point to a role for hydrophobic residues C-terminal to the LPEL residues as being important in displacing the HIF-1α_797–805_ helix.^67,72,73^ Similarly, ^15^N-relaxation and side chain methyl ^2^H-relaxation experiments on p300 and side chain methyl ^2^H-relaxation for bound HIF-1α demonstrated (i) that side-chain and backbone dynamics for p300 upon binding to CTAD-HIF-1α involve an unfavourable conformational entropy change on complex formation (with the backbone contribution dominant), (ii) that HIF-1α similarly undergoes a significant side chain conformational entropy change upon p300 recognition and (iii) the N-terminal region of HIF-1α, the residues in p300 contacting the LPQL motif and the C-terminus of p300 remain dynamic when bound.^74^ Finally, comparative *in silico* alanine scanning results (Fig. S12, ESI†) determined using BAlaS^56^ (a web-based server version of BudeAlaScan) for HIF-1α/p300 (PDB ID: 1L8C), CITED2/p300 (PDB ID: 1P4Q) and the CITED-HIF-fusion/CBP complex (PDB ID: 7LVS) reported by Appling *et al.* show a dispersed distribution of potential hot residues (similar to that observed for HIF-1α/p300 (PDB ID: 1L8C) using BudeAlaScan), but with a greater proportion towards the N-terminus in CITED2 and the C-Terminus in HIF-1α. The variation of one of these hot residues (L63A) in the CITED2-HIF-1α fusion peptide corresponding to L822A in this work) resulted in the complete displacement of the C terminal HIF-1α_815–826_ helix which allowed the binding of the N-terminal helix of the fusion peptide (corresponding to CITED2_216–246_).^47^ This implies that although individual variations do not have a significant effect on overall binding affinity they can be important mechanistically in mediating allosteric responses. Our ITC experiments can be rationalized in the context of these data; the energetically unfavourable folding of HIF-1α (reflected by the large negative entropy) is compensated by a large number of weak interactions with the target (strongly favourable enthalpy), which can be achieved through a fuzzy interaction. In contrast, CITED2 folding seems to be less energetically unfavourable (weaker entropy and therefore less enthalpic compensation) suggesting a more ordered complex. Crucially where CITIF is concerned, the N-terminal fragment of CITED2_224–243_ is derived from a region that is highly ordered in the CITED/p300 interaction and so the observed enthalpy–entropy compensation might be excepted for CITIF which marries the N-terminus of CITED2 with the C-Terminus of HIF-1α. Overall, the results are fully consistent with the sequence variation studies described above in which variants with a significant predicted ΔΔ*G* were observed to bind with comparable affinity, less favourable enthalpy and more favourable entropy when compared to the parent sequence; this, may be attributed to HIF-1α binding in a more disordered manner (thus incurring a lower entropic cost) with concomitant loss of productive non-covalent interactions. Taken together, the results underscore recent observations on the protein–protein interactions of intrinsically disordered regions in which sequence variation has limited impact on binding affinity;^75,76^ enthalpy–entropy compensation provides the scope for such fuzzy interactions to accommodate sequence variation without significant impact on binding affinity and therefore function.

## Conclusions

We have shown using a combination of single and multiple alanine sequence variants of HIF-1α alongside sequence hybrids with the negative regulator of HIF-1α (CITED2) that interaction with p300 is highly tolerant to sequence variation as demonstrated by fluorescence anisotropy and isothermal titration calorimetry. Recent studies on the interaction of p300(CBP) with HIF-1α or CITED2 have largely focussed on dynamic structural studies and molecular dynamics simulations to rationalise the displacement of HIF-1α from p300 by CITED2.^40,46,47,53,67,72–74^ Our equilibrium measurements for a range of sequence variants provide complementary data demonstrating interaction between HIF-1α and p300 is characterized by a large favourable enthalpy and large unfavourable entropy of binding. The absence of dramatic changes in binding affinity for alanine variants taken together with an observed enthalpy–entropy compensation is consistent with significant chelate co-operativity^21,50^ and dispersal of binding energy across the sequence, with binding free energy derived from non-covalent contacts between the HIF-1α backbone (in addition to side-chains) and the surface of the p300 CH1 domain, alongside favourable long range electrostatic and transient side-chain interactions during HIF-1α folding. Whilst enthalpy–entropy compensation can be attributed to multiple factors^62–66^ as we acknowledge earlier, such behaviour provides a mechanism for the intrinsically disordered HIF-1α sequence to tolerate sequence variation by adopting a more disordered bound state in its interaction with p300. Binding of CITED2 to p300 however is characterized by small favourable enthalpy and entropy changes, yet (in our hands) its affinity for p300 is slightly higher than that of HIF-1α and therefore may also augment the allosteric changes that accompany ternary complex formation between HIF-1α, CITED2 and p300 *en route* to unidirectional displacement of HIF-1α by CITED2. Such behaviour is encompassed in CITIF, a HIF-1α-CITED2 hybrid sequence; p300 affinity is higher than HIF-1α and comparable to CITED2, with a thermodynamic signature that is intermediate between the two representing a consonance between the high affinity less dynamic CITED2 sequence and the more fuzzy HIF-1α. This and the sequence dependent competition mechanism by which the negative feedback regulator CITED2 displaces HIF-1α may provide insight to inform design of selective HIF-1α modulators. More broadly, these results underscore the advantageous features of intrinsically disordered regions in facilitating function^77,78^ whilst such sequence tolerance may represent an additional rationale for the prevalence of disease relevant mutations within intrinsically disordered regions.^79^

## Funding information

This work was supported by EPSRC (EP/N013573/1 and EP/K039202/1) and the BBSRC/EPSRC-funded Synthetic Biology Research Centre, BrisSynBio (BB/L01386X/1). This project has received funding from the European Union's Horizon 2020 research and innovation programme under the Marie Skłodowska-Curie grant agreement no. MSCA-IF-2016-749012. Z.H. received funding from the National Research, Development and Innovation Office – NKFIH PD 135324. A. J. W. wishes to acknowledge the support of a Royal Society Leverhulme Trust Senior Fellowship (SRF\R1\191087).

## Abbreviations list

ALAscan: Alanine scan
BUDE: Bristol University Docking Engine
C-TAD: Carboxy-terminal transactivation domain
FA: Fluorescence anisotropy
GFP: Green fluorescent protein
GST: Glutathione S-transferase
HIF-1α: Hypoxia-inducible factor 1-alpha
ITC: Isothermal titration calorimetry
mAV: Multiple alanine variant
NMR: Nuclear magnetic resonance
PPI: Protein–protein interaction
sAV: Single alanine variant
wt: wild type

## Conflicts of interest

The authors declare no competing financial interests.

## Supplementary Material

CB-003-D2CB00026A-s001
