# Supplementary material for: Understanding p300-transcription factor interactions using sequence variation and hybridization
Source: RSC Chem Biol. 2022 Apr 11;3(5):592–603. doi: 10.1039/d2cb00026a (PMC9092470; doi:10.1039/d2cb00026a)
Supplement: CB-003-D2CB00026A-s001 [file CB-003-D2CB00026A-s001.pdf]

Electronic Supplementary Material (ESI) for RSC Chemical Biology.
This journal is © The Royal Society of Chemistry 2022

Understanding p300-transcription factor interactions using sequence variation and hybridization

Fruzsina Hobor,[a,b] Zsofia Hegedus,[c*] Amaurys Avila Ibarra,[d,e] Vencel L. Petrovicz,[c] Gail J. Bartlett,[d,e,f] Richard B. Sessions,[d,e] Andrew J. Wilson,[a,g*] and Thomas A. Edwards[a,b*]

[a]Astbury Centre for Structural Molecular Biology, University of Leeds, Woodhouse Lane, Leeds LS2 9JT, UK

[b]School of Molecular and Cellular Biology, University of Leeds, Woodhouse Lane, Leeds LS2 9JT, UK

[c] Department of Medical Chemistry, University of Szeged, Dóm tér 8, H-6720 Szeged, Hungary

[d]School of Biochemistry, University of Bristol, Medical Sciences Building, University Walk, Bristol BS8 1TD, UK

[e]BrisSynBio, University of Bristol, Life Sciences Building, Tyndall Avenue, Bristol BS8 1TQ, UK

[f]School of Chemistry, University of Bristol, Cantock's Close, Bristol BS8 1TS, UK

[g]School of Chemistry, University of Leeds, Woodhouse Lane, Leeds LS2 9JT, UK

*e-mail: hegedus.zsofia@med.u-szeged.hu, a.j.wilson@leeds.ac.uk, t.a.edwards@leeds.ac.uk

Supporting information

Table of Contents

Supplementary Figures and Tables 3
Figure S1 Comparison of binding energy changes predicted by Robetta and BUDE 3
Figure S2 Structural visualization of predicted hot residues. 4
Figure S3 Fluorescence anisotropy direct titrations of GFP with GST-p300. 5
Figure S4 Representative fluorescence anisotropy titration data for sAV and mAV GFP-HIF-1α peptides 6
Figure S5 ITC data for the interaction of GFP-HIF-1α$_{776-826}$ variants. 6
Figure S6 Isothermal titration calorimetry data for the interaction of expressed HIF-1α$_{776-826}$, HIF-1α$_{786-826}$ CITED2$_{224-259}$, CITED2$_{216-269}$ and CITIF peptides. 7
Figure S7 Apparent K_D fits for the competition ITC experiments 8
Table S1 Apparent K_D values and thermodynamic parameters for competition ITC experiments. 9
Figure S8 Sequences of the truncated CITED2 peptides and isothermal titration calorimetry data. 10
Table S2 Thermodynamic parameters for shortened CITED2 sequences. 10
Figure S9 ITC enthalpograms used to fit cooperative parameters. 11
Table S3 Thermodynamic parameters extracted from the global fitting of the competition titrations. 11
Figure S10 Predicted titration curve for HIF-1α$_{776-826}$ titrated to CITED2$_{216-269}$/p300 14
Figure S11 Fluorescence anisotropy competition titration data for CITIF peptides 12
Figure S12 *in silico* Alanine scanning data for HIF-1α/p300 (PDB ID: 1L8C), CITED2/p300 (PDB ID: 1P4Q) and CITED-HIF-fusion/CBP.[1] 13
Table S4 Statistics obtained for the p300/CITIF co-crystal structure 14
Figure S13 Helical regions of the CITIF/p300 complex crystal structure. 15
Figure S14 Overlay of the CITED2-HIF-1α fusion peptide/CBP crystal structure with the CITIF/p300 crystal structure
Materials and Methods 16
In silico predictions 16
Plasmids for protein production 16
Expression and purification of p300 CH1 domain 16
Figure S15 Sequence and SDS gel and size-exclusion chromatogram of purified p300 CH1 18
Figure S16 Size exclusion chromatogram and SDS gel of purified GFP-HIF-1α
Expression and purification of GFP tagged HIF-1α and GFP-HIF-1α alanine variants 19
Expression and purification of untagged HIF-1α, CITED2 and CITIF 20
Peptide synthesis and purification 21
Fluorescence anisotropy 23
Isothermal titration calorimetry 24
Table S5 Concentrations used in competition ITC experiments. 25
Figure S17 Arrow diagram of the model used to fit ITC data to obtain the apparent K_D values 26
Figure S18 Diagram of the model used to fit the ITC data to obtain cooperative parameters 26
Co-crystallization 28
Table S6 Oligonucleotide sequences used for site-directed mutagenesis 29
Peptide characterization data 30
References 41

Supplementary Figures and Tables

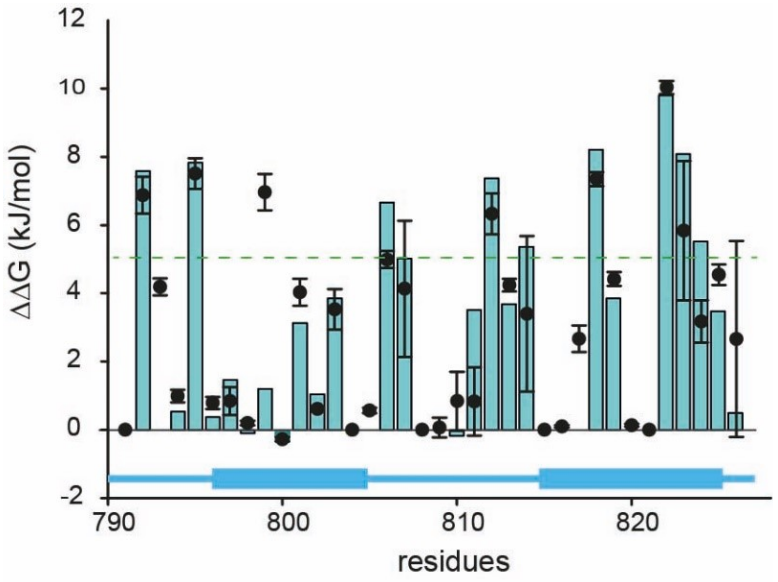

Figure S1 Comparison of binding energy changes predicted by Robetta (cyan bars) and BUDE (black circles) for the HIF-1α/p300 interaction. For Robetta the lowest energy structure was used from the NMR derived ensemble PDB ID: 1L8C. For BUDE the 20 lowest energy structures from the NMR ensemble used in the prediction, circles denote average predicted ΔΔG, error bars the standard deviation.

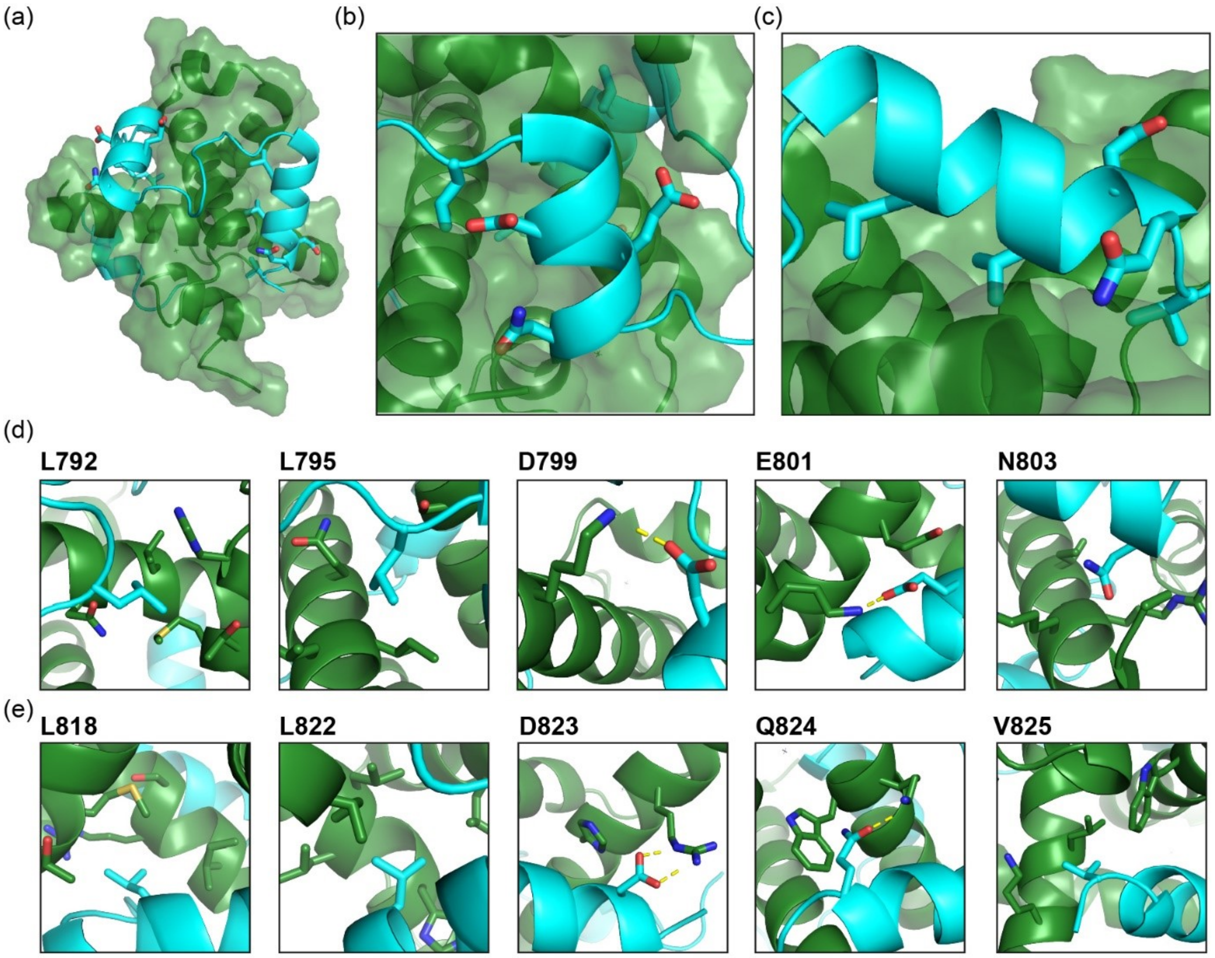

Figure S2 Structural visualization of predicted hot residues. (a) surface representation of p300 (green) bound to HIF-1α (cyan ribbon) highlighting the C-terminal helices (HIF-1α$_{797-805}$ and HIF-1α$_{815-826}$); (b) hot residues found in the HIF-1α$_{797-805}$ helix; (c) hot residues found in the HIF-1α$_{815-826}$ helix; (d) intermolecular interactions of individual hot-residues from HIF-1α$_{797-805}$ helix (e) intermolecular interactions of individual hot-residues from HIF-1α$_{815-826}$ helix (HIF-1α is shown in cyan, p300 in green and selected hotspots are shown as stick representation, polar contacts indicated with yellow dashed lines)

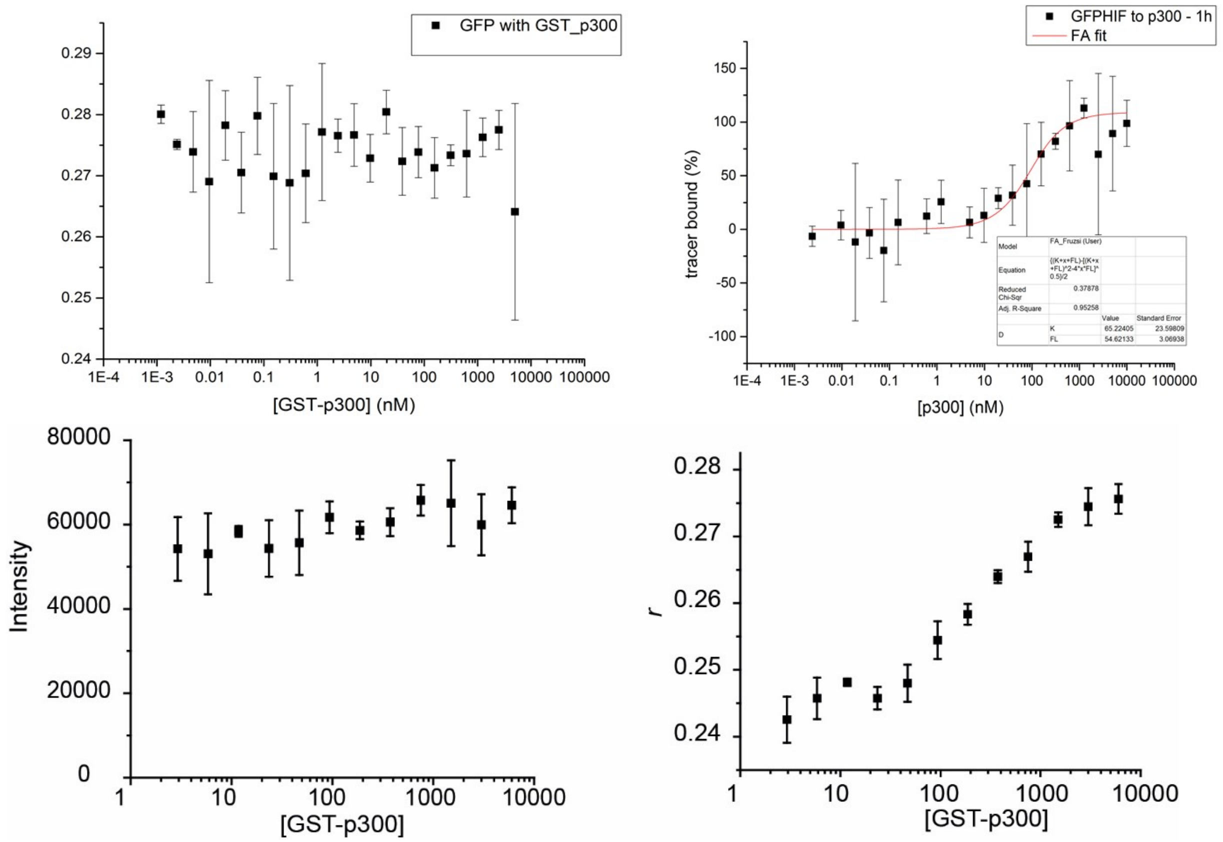

Figure S3 Fluorescence anisotropy direct titrations of 50nM GFP with increasing concentration of GST-p300 (top left) and GFP-HIF-1α$_{776-826}$ with p300 (top right) to show that GFP does not interact with GST-p300 nor does the presence of GST affect the binding to GFP-HIF-1α. Bottom left shows intensity and bottom right shows raw anisotropy as a function of GST-p300 for titration against GFP-HIF-1α with he corresponding fraction bound data including in Figure 2 for this titration (25 mM Tris-HCl, 150 mM NaCl, 1mM DTT, pH 7.4, starting from 10μM p300 concentration using a two-fold serial dilution series across the plate). The data was analysed using Origin. Error bars represent standard deviation of technical triplicates.

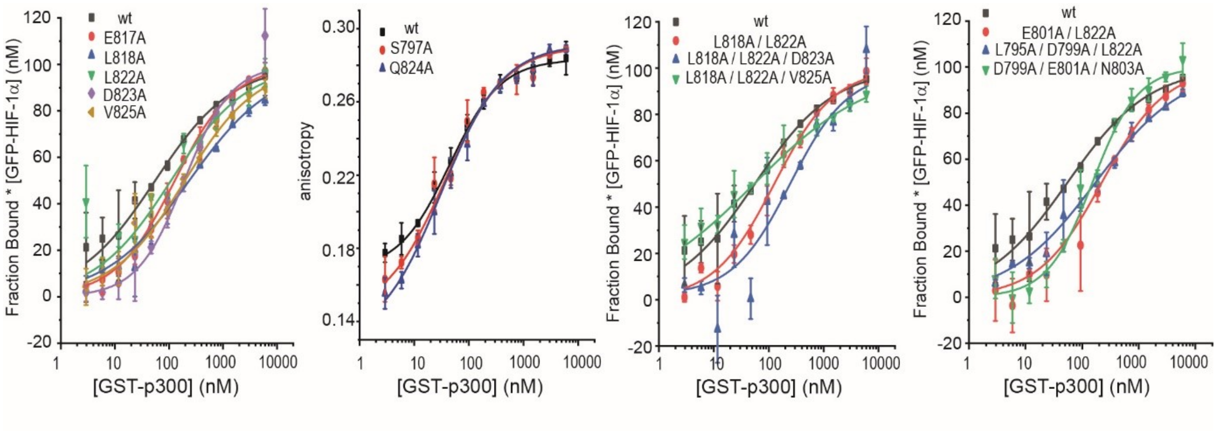

Figure S4 Representative fluorescence anisotropy titration data for sAV and mAV GFP-HIF-1α peptides interacting with GST-p300 (25 mM Tris-HCl, 150 mM NaCl, 1mM DTT, pH 7.4). Error bars represent standard deviation of technical triplicates.

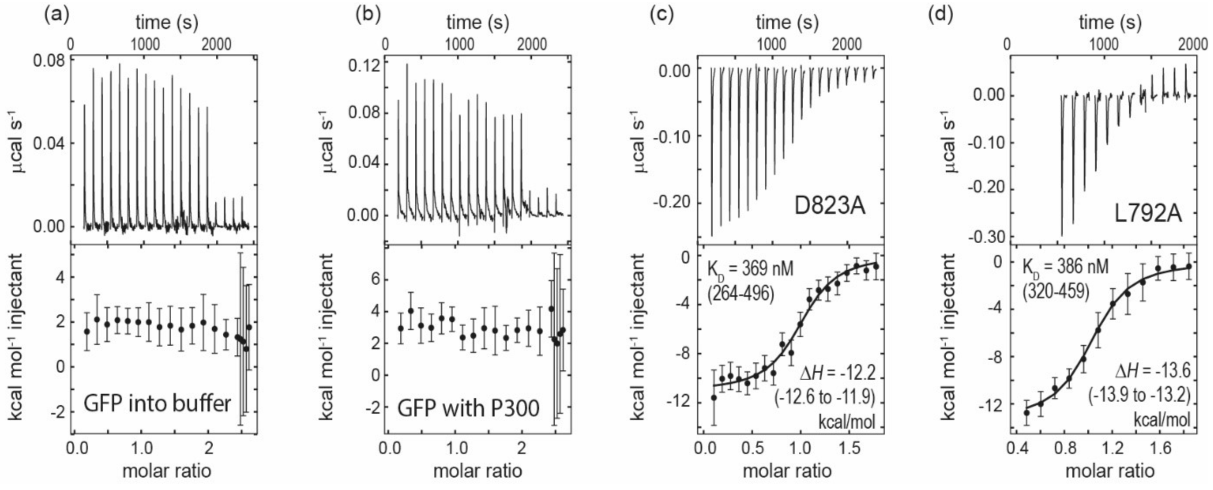

Figure S5 Heat of dilution of (a) GFP titrated to buffer, (b) GFP titrated to p300 and (c) Raw ITC (upper) data and fitted thermogram (lower) for the interaction of GFP-HIF-1α$_{776\text{-}826}$ D823A variant (37°C in 25 mM Tris-HCl, 150 mM NaCl, 1mM DTT, pH 7.4) using 10 μM p300 in the cell and 100 μM HIF-1α variant in the syringe. ITC experiments were carried out using a Microcal ITC200i instrument (Malvern). All proteins were dialysed against the same buffer prior to experiment, buffer or 10 μM p300 was present in the cell and titrated with 100 μM GFP-HIF-1α or GFP loaded into the syringe using 16 x 2 μL injections with 120 s spacing between the injections. Heats of GFP or GFP-HIF-1α dilution were subtracted from the measurement raw data. Error bars represent the estimated integration errors.

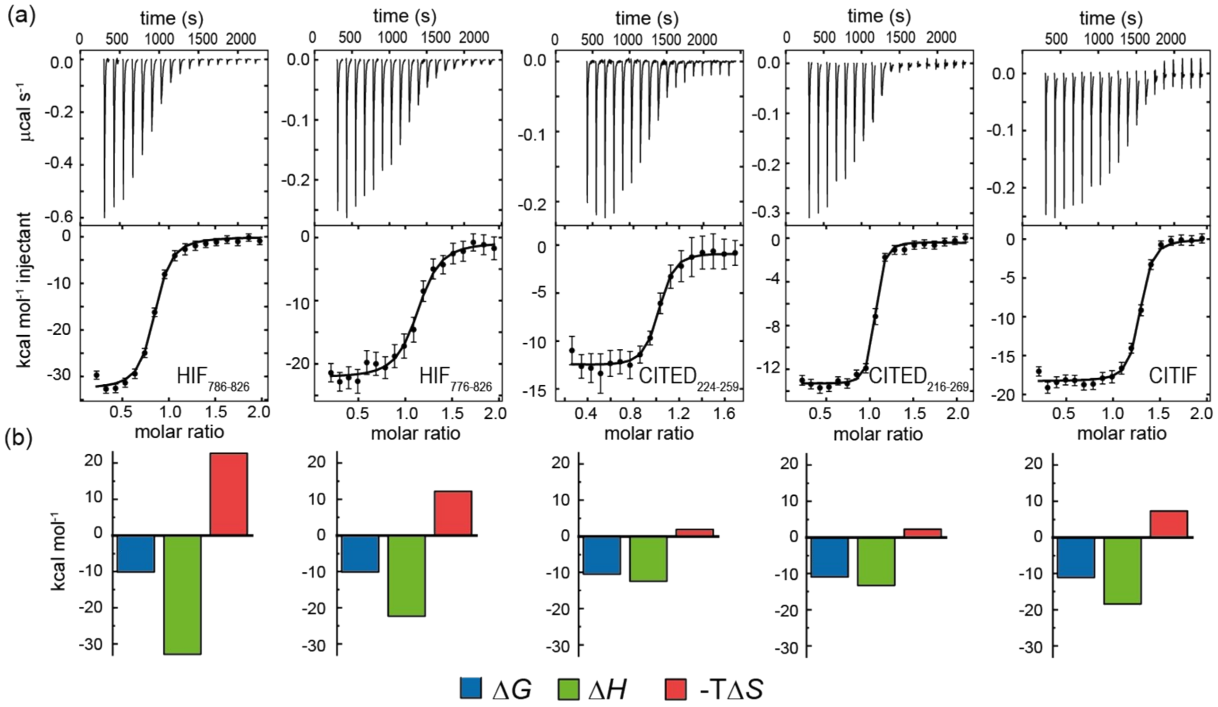

Figure S6 Isothermal titration calorimetry data for the interaction of expressed HIF-1α$_{776\text{-}826}$, HIF-1α$_{786\text{-}826}$ CITED2$_{224\text{-}259}$, CITED2$_{216\text{-}269}$ and CITIF peptides with p300. (a) Raw ITC (upper) data and fitted thermogram (lower) (40 mM sodium phosphate, pH 7.5 100 mM NaCl, 1 mM DTT buffer using 5 μM protein in the cell and 60 μM ligand in the syringe at 35°C); (b) Thermodynamic signatures for each interaction. Error bars represent the estimated integration errors.

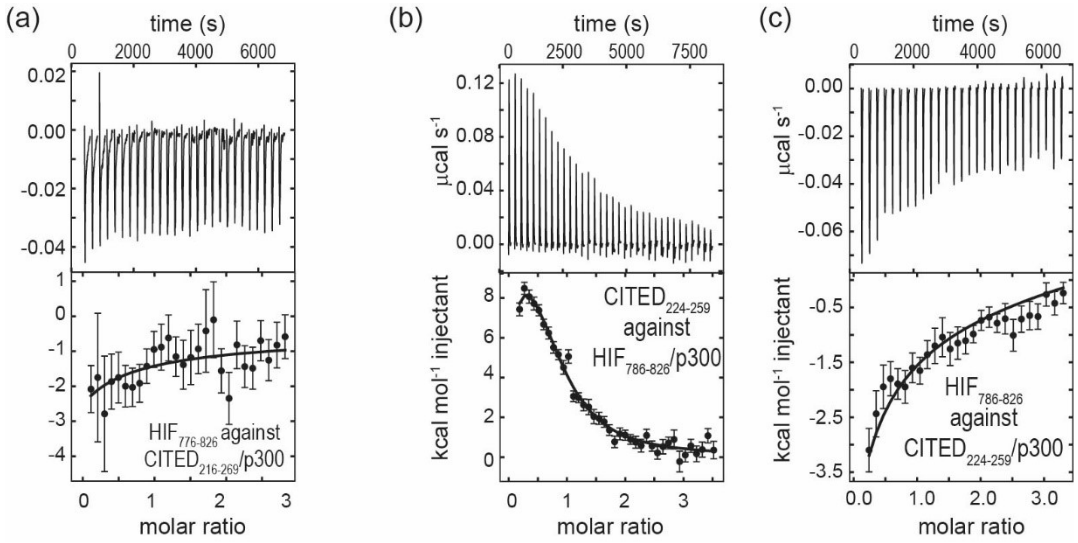

Figure S7 Apparent K_D fits for the competition ITC experiment using a competition interaction model. p300/ligand complexes were prepared by titrating the appropriate ligand first to p300 in the cell using 3-5 μM protein concentration, the titration stopped when it reached close to saturation conditions, which resulted in 1.15-1.5 excess of ligand compared to p300 in the cell (Table S5) Then it was titrated with the competitor ligand using 30-60 μM concentration in the syringe, 5 or 10 uL injections with 180-240 s spacing. Background titrations were performed without p300 in the cell and subtracted from the measurements. Apparent $K_{D,app}$ and ΔH_{app} values data were fitted to a competition model (Fig. S17) in SEDPHAT. Error bars represent the estimated integration errors.

Table S1 Apparent K_D values and thermodynamic parameters for competition ITC experiments derived from fitting the data to a competition model depicted in Fig. S17. 68% confidence intervals of the binding parameters are shown in brackets. Raw data are presented in Fig. 5e and Fig. S7.

	CITED2 titrated to preformed p300/HIF-1α complexes		
	$K_{D,app\ CITED2}$, nM	$\Delta H_{app,\ CITED2}$, kcal/mol	$\Delta S_{app,\ CITED2}$, cal/mol/K
CITED2$_{216-269}$ titrated to HIF-1α $_{776-826}$/p300 complex[a]	1.1 (0.9 – 1.4)	- 14.99 (-15.22 to -14.77)	-7.69
CITED2$_{224-259}$ titrated to HIF-1α $_{776-826}$/p300 complex[a]	12.2 (11.2 – 13.1)	-13.44 (-13.57 to-13.31)	-7.44
CITED2$_{216-248}$ titrated to HIF-1α $_{776-826}$/p300 complex[a]	46.9 (39.6 – 55.6)	-13.05 (-13.64 to -12.41)	-8.83
CITED2$_{224-259}$ titrated to HIF-1α $_{786-826}$/p300 complex[b]	8.4 (7.7 – 9.1)	-14.13 (-14.34 to -13.91)	-8.92
	HIF-1α titrated to preformed p300/ CITED2 complexes		
	$K_{D,app\ HIF\text{-}1\alpha}$, nM	$\Delta H_{app,\ HIF\text{-}1\alpha}$, kcal/mol	$\Delta S_{app,\ HIF\text{-}1\alpha}$, cal/mol/K
HIF-1α$_{776-826}$ titrated to CITED2$_{216-259}$ /p300 complex[c]	695 (277 – 2050)	-26.37 (< -30 to -20.6)	-57.4
HIF-1α $_{786-826}$ titrated to CITED2$_{224-259}$ /p300 complex[d]	44.57 (31.39 – 63.58)	-22.3 (-23.99 to -20.99)	-38.8

[a] Fitted using fixed K_D = 52.5 nM and ΔH = -22.9 kcal/mol for **HIF-1α$_{776-826}$**
[b] Fitted using fixed K_D = 42.9 nM and ΔH = -25.8 kcal/mol for **HIF-1α $_{786-826}$**
[c] Fitted using fixed K_D = 26.3 nM and ΔH = 13.2 kcal/mol for **CITED2$_{216-259}$**
[d] Fitted using fixed K_D = 9.1 nM and ΔH = -12.4 kcal/mol for **CITED2$_{224-259}$**. [c-d]These values should be treated with caution due to small heat change during the titrations. (See Figure S7.)

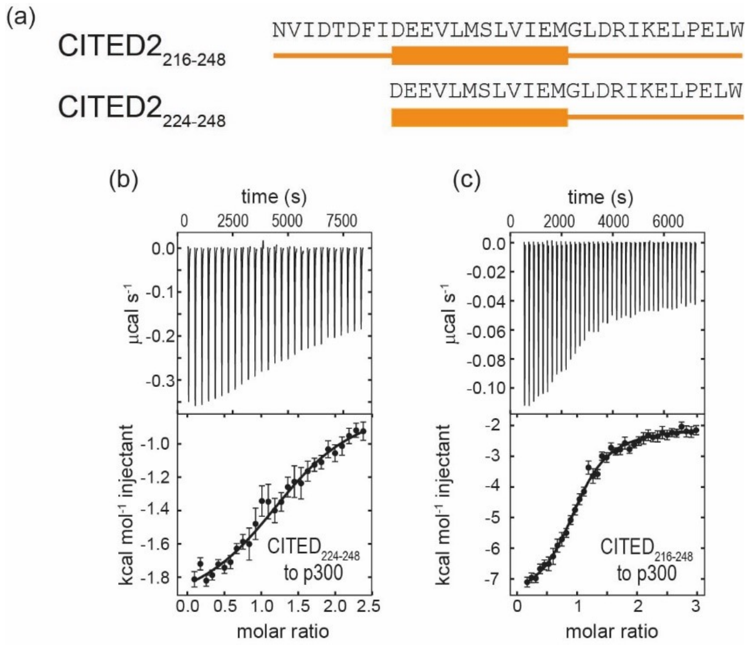

Figure S8 (a) Sequences of the truncated CITED2 peptides and isothermal titration calorimetry data for (b) $CITED_{224\text{-}248}$ titrated to p300 using 45 μM p300 in the cell and 500 μM peptide in the syringe and (c) $CITED_{216\text{-}248}$ titrated to p300 using 5 μM p300 in the cell and 50 μM peptide in the syringe. All measurements were carried out 40 mM sodium phosphate, pH 7.5 100 mM NaCl, 1 mM DTT buffer at 35°C. Error bars represent the estimated integration errors.

Table S2 Thermodynamic parameters for shortened CITED2 sequences. 68% confidence intervals for the fitting are shown in brackets. Data fitted to a single binding site model, fixed 1:1 stoichiometry with incompetent protein fraction as an adjustable parameter.

	K_{D}, μM	ΔH kcal mol^{-1}	ΔS cal mol^{-1} K^{-1}
**$CITED2_{224\text{-}248}$**	10.44 (7.86 – 14.2)	-1.28 (-1.45 to -1.15)	18.62
**$CITED2_{216\text{-}248}$**	0.303 (0.230 – 0.397)	-5.7 (-6.1 to -5.4)	11.3

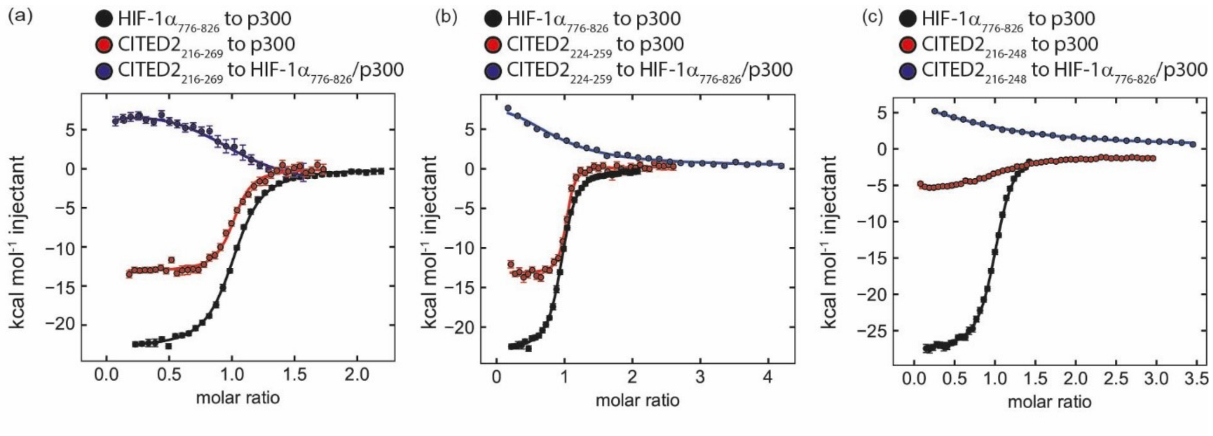

Figure S9 ITC enthalpograms used to fit cooperative parameters (α and ΔΔ*H*) using the global fitting procedure to a model where the ternary CITED2-HIF-1α-p300 complex formation is involved for measurements with a) $CITED2_{216-269}$ b) $CITED2_{224-259}$ and c) $CITED2_{216-248}$ titrated to p300 (red curve) and to p300/HIF-$1α_{776-826}$ complex (blue curve) including a HIF-1 $α_{776-826}$ to p300 titration (black curve) for all fitting. Fitting was performed using a model depicted in Fig. 5e and Fig S18. Conditions as in Fig S7-8 and Table S5.

Table S3 Thermodynamic parameters extracted from the global fitting of the competition titrations, using titrations shown in Fig S9. with a model including a ternary complex (CITED2-HIF-1α-p300) formation (Fig 5e and Fig S18.) α is the cooperativity constant from which ΔΔG was derived using Equation 15, which is the additional Gibbs energy due to cooperative interactions when CITED2 binds to the preformed p300/HIF-$1α_{776-826}$ complex compared to the CITED2/p300 interaction. 68% confidence intervals of the fitted values are shown in brackets.

		K_D,nM	Δ*H* kcal mol^{-1}	Δ*S* cal mol^{-1} K^{-1}	α	ΔΔG kcal mol^{-1}	ΔΔH kcal mol^{-1}
1	**$CITED_{224-259}$**	11.6 (9.4 - 14.5)	-13.4 (-13.6 to -12.3)	-7.2	1*10^{-6} (<1*10^{-6} - 3*10^{-4})	8.44 (> 8.5 to 4.88)	-0.09 (-1 to 1)[a]
	**$HIF_{776-826}$**	48.8 (38.4 - 56.3)	-22.7 (-23.1 to -22.4)	-40.3			
2	**$CITED_{216-269}$**	26.9 (33.8 – 21.4)	-13.2 (-13.4 to -13.0)	-8.3	0.6 (0.9 -1.75)	0.31 (-0.34 to 0.9)	20.5 (19.9 - 21.2)
	**$HIF_{776-826}$**	52.3 (64.2 – 41.2)	-22.9 (-23.4 to -22.5)	-41.1			
3	**$CITED2_{216-248}$**	297 (238 – 373)	-5.7 (-5.9 to -5.4)	11.3	0.12 (0.06 – 0.9)	1.27 (0.9 – 1.69)	9.8 (8.5 – 11.3)
	**$HIF_{776-826}$**	67 (59 – 75)	-27.8 (-28.1 to -27.5)	-57.4			

[a] fitted with a constraint from -1 to 1.

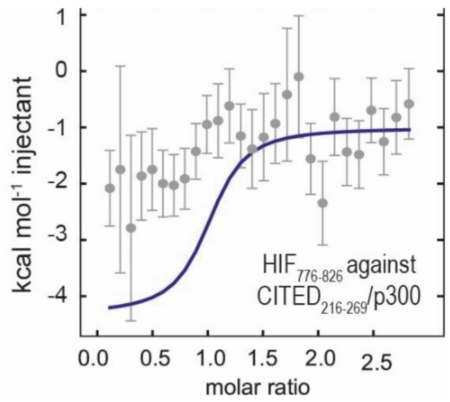

Figure S10 Predicted titration curve (blue) using the fitted parameters in Table S3 for the titration where HIF-1α$_{776\text{-}826}$ is titrated against preformed CITED2$_{216\text{-}269}$/p300 (raw data is shown in Fig S7a). The fitting model is the same as in Fig. S9 and Table S3.

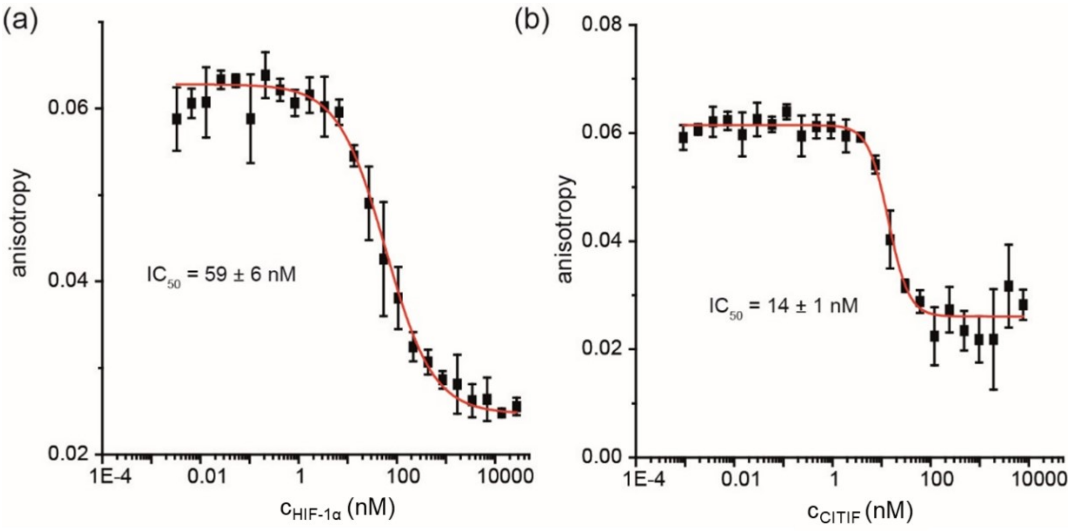

Figure S11 Fluorescence anisotropy competition titration data for: (a) HIF-1α$_{786\text{-}826}$ and (b) CITIF peptides illustrating that CITIF competes with HIF-1α for p300 (25 mM Tris-HCl, 150 mM NaCl, 1mM DTT, pH 7.4 using 5 nM FAM-HIF$_{786\text{-}826}$ as tracer, with a two-fold dilution series of the competitor ligand starting from 10 μM over 24 points); (note: whilst the FA competition experiments for the expressed peptides revealed some differences in IC_{50} values that are consistent with a higher affinity of CITIF than HIF-1α for p300 the concentrations of HIF-1α and p300 in the assay are such that inhibition occurs at the limit of the assay, thus these numbers should be treated with caution). Error bars represent standard deviation of technical triplicates

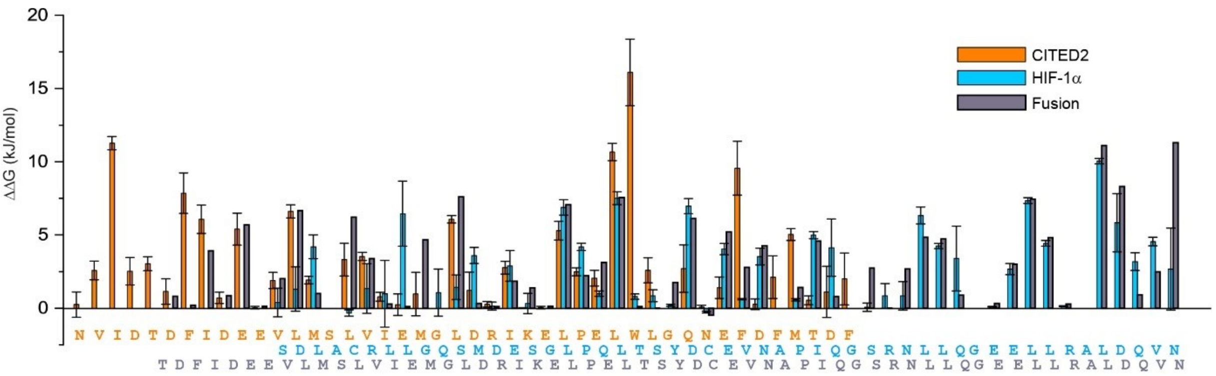

Figure S12 *in silico* Alanine scanning data were calculated for HIF-1α/p300 (PDB ID: 1L8C), CITED2/p300 (PDB ID: 1P4Q) and CITED-HIF-fusion/CBP (PDB ID: 7LVS) structures using the BAlaS server (https://pragmaticproteindesign.bio.ed.ac.uk/balas/).[1]

Table S4 Statistics obtained for the p300/CITIF co-crystal structure

**Reservoir conditions**	0.1 M HEPES pH 6.5, PEG 6K 35%
**Data Collection**	
X-ray source	DLS Beamline i04
Processed using	xia2 (DIALS, Aimless)
oscillations	0.1
images collected	3600
Space group	$P\ 1\ 2_1\ 1$
**Unit cell dimensions**	
a, *b*, *c*, (Å)	30.50, 48.60, 39.83
α, β, γ (°)	90, 103.87, 90
Resolution	2.0 - 29.6 (2.0 - 2.07)
Observations	51028 (2141)
Unique reflections	7688 (747)
R_{merge} (I)	0.109 (0.744)
R_{meas} (I)	0.119 (0.814)
R_{pim} (I)	0.047 (0.325)
CC 1/2	0.991 (0.870)
I/σ	7.5 (2.9)
Completeness	99.7 (96.5)
Redundancy	6.6 (6.0)
**Refinement**	
Protein molecules in au	2
Rwork/Rfree	0.221/0.245
No atoms	1210
Protein	1166
Ligand	3 (Zn)
Water	41
**Mean B factors (Å)**	
Protein	79.14
Ligand	57.35
Water	77.69
**R.m.s. deviations**	
Bond length (Å)	0.016
Bond angles	1.55
**Ramachandran statistics**	
% favoured	95.14
% allowed	4.86
% outliers	0
Clashscore	13.3

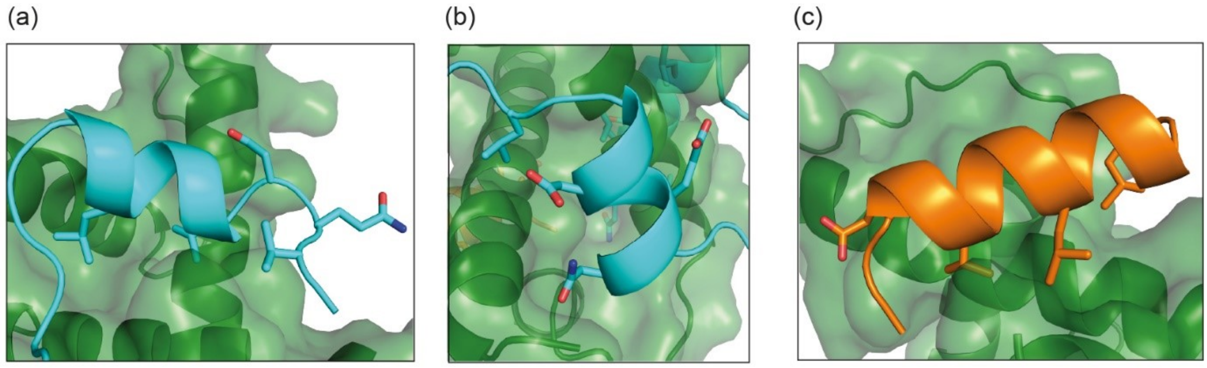

Figure S13 Helical regions of the CITIF/p300 complex crystal structure (PDB: 7QGS), hot residues are shown as stick representation. (a) Residues corresponding to HIF-1α$_{815\text{-}826}$ helix (b) residues corresponding to HIF-1α$_{797\text{-}805}$ helix (c) residues corresponding to helix CITED2$_{224\text{-}237}$ helix.

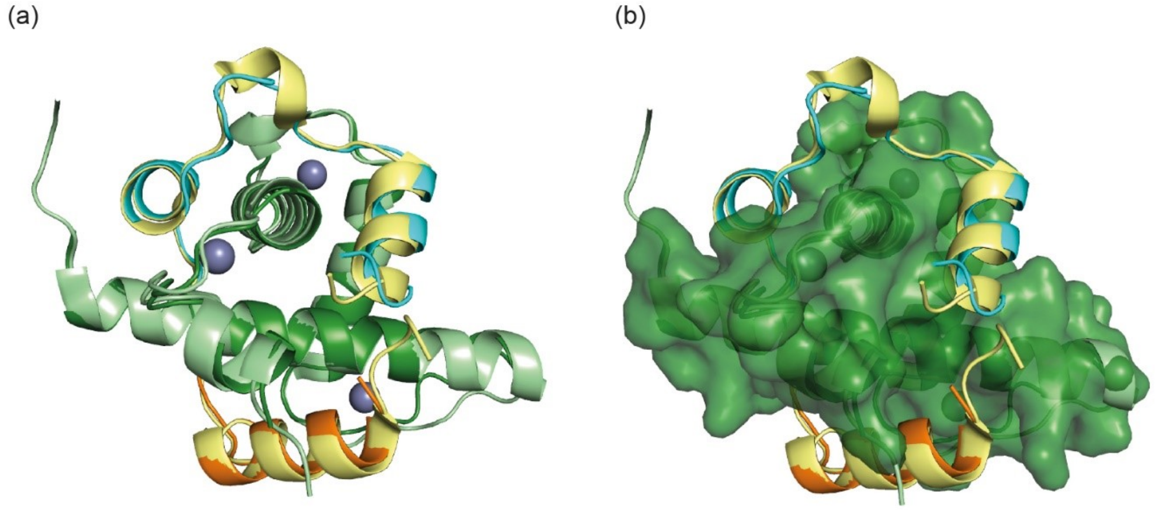

Figure S14 Overlay of the CITED2-HIF-1α fusion peptide/CBP crystal structure (PDB:7LVS, CBP in light green, fusion peptide in yellow) with the CITIF/p300 crystal structure (PDB:7QGS) p300 in green, CITIF in cyan/orange (cyan corresponding to HIF-1α$_{792\text{-}826}$ and orange corresponding to CITED2$_{224\text{-}243}$ residues). Only minor differences are observed between the two structures, with the only notable conformational difference observed for Gln39-Leu44 (7QGS numbering), whereby a more linear conformation is adopted in 7QGS.

Materials and Methods

In silico predictions

Alanine scan using Robetta

Alanine scanning data for HIF-1α/p300 were calculated with the Robetta server, http://robetta.bakerlab.org, using the lowest energy structure from the NMR derived ensemble PDB ID: 1L8C.

BUDE Alanine scan

Alanine scanning data (show in Fig. 1g) for HIF-1α /p300 were calculated using BudeAlaScan as previously described[1] using the NMR derived ensemble PDB ID: 1L8C (standard deviations represent the variation in determined ΔΔG between individual structures in from the ensemble). Additionally alanine scanning data were calculated for the HIF-1α/p300, CITED2/p300 and CITED-HIF-fusion/CBP structures using PDB ID: 1L8C, 1p4q and 7lvs respectively using the BalaS server (https://pragmaticproteindesign.bio.ed.ac.uk/balas/).[1]

Plasmids for protein production

The DNA sequence of human p300 (UniProtKB - Q09472) containing the CH1 domain (Zinc finger, TAZ type 1) from residues 330-420 was cloned into pGEX-6P-2 or pGEX-4T-1 plasmids using BamHI/XhoI restriction sites.

The genes encoding *H sapiens* HIF-1α (residues 776-826 and 786-826, Uniprot Q16665), CITED2 (residues 224-255 and 216-269, Uniprot Q99967) and CITIF (containing CITED2 224-243 fused with HIF-1α residues 792-826) were inserted into pET28:GFP plasmid using BamHI/XhoI restriction sites creating His_{10}-GFP constructs. HIF-1α mutants (residues 776-826) were produced using the Quikchange Site Directed Mutagenesis Kit (Agilent) and the wild type protein expression vector as template (oligo sequences are shown in Table S5). The sequences of all constructs were confirmed by DNA sequencing prior to expression.

Expression and purification of p300 CH1 domain

Expression and purification of GST-p300 was performed as described previously[2] using either pGEX-6P2 or pGEX-4T1 plasmids. *E. coli* strain Rosetta 2 (transformed with pGEX-6P2-p300) or BL21 E.Coli pLysS (transformed with pGEX-4T1-p300) were grown at 37°C to OD600 0.6-0.8 and induced with 0.1 mM IPTG and 50 μM $ZnSO_4$ was added and incubated overnight at 18 °C. Cells were harvested and lysed by sonication and centrifuged at 25.000 g for 30 minutes at 4ºC. The supernatant was applied to glutathione beads (Glutathione Sepharose 4B, GE

Healthcare) and washed with 20 mM Tris, pH 8, 150 mM NaCl and 20 mM Tris, pH 8, 500 mM NaCl. GST was cleaved on-column overnight at 4ºC using PreScission protease (pGEX-6P-2 plasmid) or Thrombin protease (pGEX-4T-1 plasmid). For the fluorescence anisotropy (FA) experiments the GST tag cleavage step was omitted and the GST tagged protein was eluted using 20 mM glutathione containing buffer. The eluted fractions were concentrated and purified by size-exclusion chromatography on S75 26/60 pg column in 20 mM Tris, 150 mM NaCl, 5% glycerol, 1mM DTT pH 7.5 buffer. Collected fractions were analysed by SDS-PAGE and pure fractions were concentrated. Pure protein was analysed by high resolution mass spectrometry: For the PreScission cleaved construct: expected m/z: 10870.63 measured m/z: 10864.2471. This construct was used to perform ITC experiments on MicroCal iTC200 instrument (titrated with expressed interaction partners). For thrombin cleaved construct expected m/z: 10603.30, measured m/z: 10603.41, this construct was used to perform experiments on Microcal VP ITC (titrated with synthetic interaction partners). Concentration was determined by UV-VIS spectroscopy using 5500 M^{-1} cm^{-1} extinction coefficient.

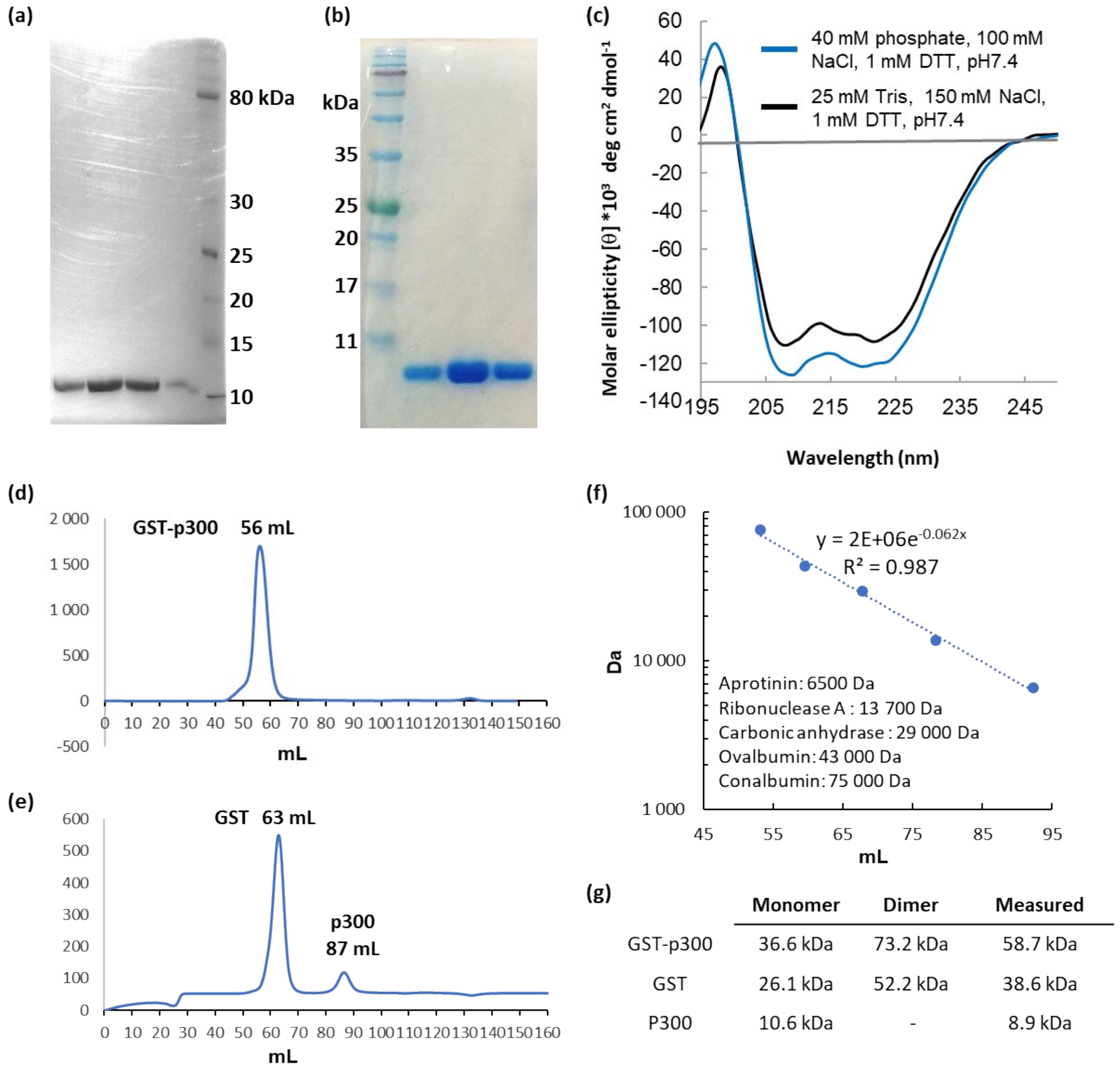

	Monomer	Dimer	Measured
GST-p300	36.6 kDa	73.2 kDa	58.7 kDa
GST	26.1 kDa	52.2 kDa	38.6 kDa
P300	10.6 kDa	-	8.9 kDa

Figure S15 SDS gel of purified p300 CH1 using a) pGEX-6P-2 plasmid followed by PreScission protease cleavage and b) using pGEX-4T-1 plasmid and thrombin cleavage. c) CD spectra of p300 CH1 in two different buffers. Size-exclusion chromatograms of d) GST-p300 and e) GST-p300 followed by thrombin cleavage on S75 26/60 pg column in 20 mM Tris, 150 mM NaCl, 5% glycerol, 1mM DTT pH 7.5 buffer. f) Calibration curve of S75 26/60 pg column using the standard protein solutions indicated on the graph. g) Comparison of expected and measured protein molecular weights. The measured molecular weight difference between GST-p300 and GST (20 kDa, which corresponds to two p300 molecules) indicates the presence of dimeric forms.

Expression and purification of GFP tagged HIF-1α and GFP-HIF-1α alanine variants

GFP-HIF-1α residues 776-826 and its alanine variants were expressed in the *E. coli* strain Rosetta 2. Bacterial cultures were grown at 37°C to a density of OD_{600} = 0.5-0.8 and protein expression then induced by addition of 1 mM isopropyl-β-D- thiogalactoside (IPTG). Upon induction, cultures were cooled and maintained at 22°C for protein expression overnight. Cell pellets were harvested by centrifugation for 7 minutes at 8655 x g and then re-suspended in lysis buffer (25 mM Tris-HCl pH 8, 500 mM NaCl) containing (1 mg mL^{-1} lysozyme, 10mg mL^{-1} of DNase1 and 1 tablet per 1 L culture protease inhibitor tablets (Complete Mini, Roche) and lysed by sonication. The cell lysate was then centrifuged for 45 minutes at 23655 x g, the supernatant was collected and applied onto a Ni-Sepharose resin column (bed size 5 mL). The column was washed with 40 mL wash buffer I (25 mM Tris-HCl, 500 mM NaCl, pH 8), 40 mL wash buffer II (25 mM Tris-HCl, 15 mM Imidazole, 500 mM NaCl, pH 8) and 400mL was buffer III (25 mM Tris-HCl, 50 mM Imidazole, 500 mM NaCl, pH 8). Bound proteins were eluted with 10 mL of elution buffer (25 mM Tris-HCl, 400 mM Imidazole, 500 mM NaCl, pH 8). The elution fraction was loaded on Superdex75 (26/600) prep grade size exclusion chromatography column, equilibrated with Gel filtration buffer (25 mM Tris-HCl, 150 mM NaCl, 1mM DTT, pH 7.4). Samples of the fractionated eluent were applied to 15% SDS gels to assess sample purity and the purified protein was concentrated using Amicon-15 10K (Merck) centrifugal concentrators with a MWCO of 10kD and stored at -80°C in small aliquots.

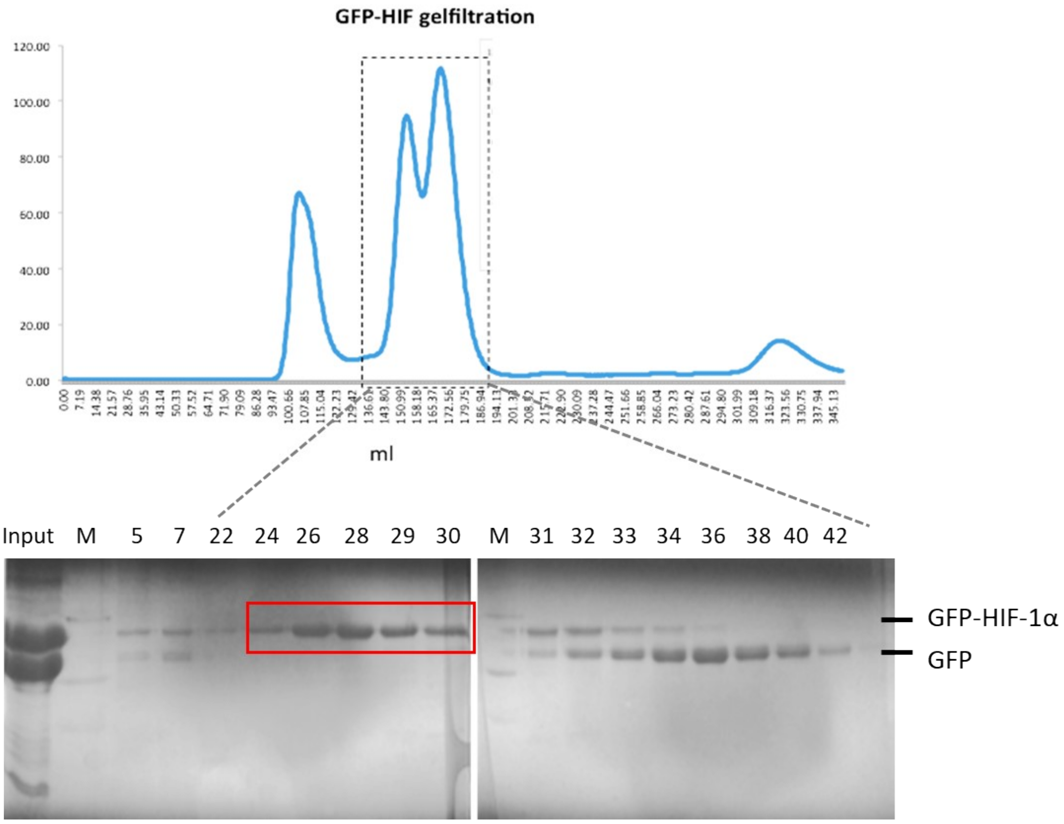

Figure S16 Purification of recombinantly expressed GFP-HIF-1α$_{776\text{-}826}$ by size exclusion chromatography. During the initial purification step the fusion-protein is partially cleaved, but the cleaved GFP could be separated from GFP-HIF-1α by size-exclusion chromatography (red square), and later the protein remained stable.

Expression and purification of untagged HIF-1α, CITED2 and CITIF

Untagged HIF-1α (residues 776-826 and 786-826), CITED2 (residues 224-255 and 216-269) and CITIF were expressed using the same conditions as described above with the following modifications. After cell lysis the inclusion bodies were washed with lysis buffer and the insoluble pellet was resuspended in 6M urea, 0.5 M NaCl, 5 mM imidazole, 50 mM Tris pH 8, 1 mM DTT and sonicated and centrifuged as before. The supernatant was applied to a His-trap HP column (5ml) and washed with 10 column volumes of the resuspension buffer, then with 4 M urea, 0.5 M NaCl, 20 mM imidazole, 20 mM Tris, pH 8, 1 mM DTT, followed by washing with 2 M urea, 0.25 M NaCl, 20 mM imidazole, 20 mM Tris, pH 8, 1 mM DTT then 20 mM Tris, 0.1 M NaCl, pH 8, 1mM DTT. PreScission protease was added to the last buffer and 5 ml applied to the column and on-column cleavage performed at 4°C. Cleavage was followed by HRMS. The eluted protein without further concentration, was immediately purified using RP-HPLC on an Agilent 1260 Infinity HPLC equipped with a diode array detector using a

Kinetex EVO C18 column. Eluent A: H2O + 0.1% TFA, B: ACN + 0.1 % TFA. Gradient timetable: 0 min 5% B, 10 min 45% B, 25 min: 75% B, at a 10 ml/min flow rate. The collected fractions were freeze dried and analysed using high resolution mass-spectrometry (See peptide characterization data.)

Peptide synthesis and purification

Fmoc amino acids, Diisopropylcarbodiimide and Oxyma (Ethyl cyano(hydroxyimino)acetate) were purchased from Fluorochem. DMF (HPLC grade) and ACN (HPLC grade) was purchased form VWR, piperazine from Molar Chemicals.

Peptides were synthesized using a microwave assisted automated peptide synthesizer (CEM Liberty Blue). Preparative RP-HPLC was carried out on JASCO PU-4180 system equipped with a diode array detector (MD-4015) and an automatic fraction collector (Advantec, CHF122SC). Fraction collection was monitored and programmed using ChromNAV software. HPLC-MS measurements were carried out on a Dionex Ultimate 3000 HPLC system equipped with a diode array detector and interfaced to an LTQ XL (Thermo Scientific) ion trap mass spectrometer. Analytical UV measurements were carried out on a Shimadzu LC-20AD equipped with a SPD20A UV detector. Eluents for HPLC-UV: A: 0.1% TFA/H_2O; B: 0.1% TFA/ACN. Eluents for HPLC-MS: A: 0.1% HCOOH/H_2O; B: 0.1% HCOOH/ACN.

Peptides were synthesized on a 0.1 mmol scale on Tentagel R RAM resin (resin loading 0.19 mmol/g, Iris Biotech). Couplings were performed using 5 equivalent amino acid excesses using DIC and Oxyma as coupling reagents, dissolved in DMF. Required amounts were calculated using the built-in reagent calculator of Liberty Blue. All amino acids were double coupled using high swelling (HS) Liberty Blue methods. Deprotection solution contained 10% (w/v) piperazine dissolved in 10% absolute ethanol/NMP mixture, which effectively inhibited aspartimide formation.

Cycles for automated peptide synthesis

Resin swelling (HS): DMF (15 mL), 5 minutes

Coupling cycle

Deprotection: 4mL, Microwave method: Standard Deprotection (Bubble: 2s on, 3s off; 75°C 175 W 15s, 90°C 30W 50s)

4x Wash: 4 mL

2x Coupling: Amino acid 2.5 mL, Activator 1 mL, Activator Base 0.5 mL, manifold wash volume 2 mL. Microwave method: Standard coupling (Bubble 2s on, 3s off; 75 °C 183 W 15s; 90°C 30W 110s)

2x Wash: 4 mL

Arginine coupling

Deprotection: 4mL, 75°C Initial Deprotection (Bubble 2s on, 3s off; 75°C 40W 30s)

Deprotection: Microwave method: Standard Deprotection (Bubble: 2s on, 3s off; 75°C 175 W 15s, 90°C 30W 50s)

4x Wash: 4 mL

Coupling: Amino acid 2.5 mL, Activator 1 mL, Activator Base 0.5 mL, manifold wash volume 2 mL. Microwave method: Bubble 2s on, 3s off; 25 °C 0 W 1500s; 75°C 30W 120s

Wash: 4 mL

Coupling: 75°C Coupling, Bubble 2s on, 3s off; 75°C 30W 300s

2x Wash: 4 mL

Cysteine coupling

Deprotection: 4mL, 75°C Initial Deprotection (Bubble 2s on, 3s off; 75°C 40W 30s)

Deprotection: 75°C Deprotection, Bubble 2s on, 3s off; 75°C 40W 180s

4x Wash: 4 mL

Coupling: Amino acid 2.5 mL, Activator 1 mL, Activator Base 0.5 mL, manifold wash volume 2 mL. Microwave method: 50°C coupling: 25°C 0W 120 s; 50°C 35W 240s

Coupling: Amino acid 2.5 mL, Activator 1 mL, Activator Base 0.5 mL, manifold wash volume 2 mL. Microwave method: 50°C coupling: 25°C 0W 120 s; 50°C 35W 240s

2x Wash: 4 mL

Final deprotection

Deprotection: 4 mL, Microwave method: Standard Deprotection (Bubble: 2s on, 3s off; 75°C 175 W 15s, 90°C 30W 50s)

4x Wash

Acetylation

Peptides were acetylated using 10 equivalents of acetic anhydride and DIPEA in DCM:DMF 1:1 in a fritted SPE tube, for 20 minutes 2 times at room temperature.

Cleavage

Peptides were cleaved using TFA:DTT:TIS:H_2O mixture (88:5:2:5) for 3 hours, after which TFA was evaporated and the crude peptide was precipitated in ice-cold diethyl-ether. The precipitate was washed with ether then redissolved in ACN:H_2O mixture and lyophilized.

Peptide purification

The following eluents were used for all preparative HPLC purifications and HPLC-UV methods: A: 0.1% TFA/H_2O; B: 0.1% TFA/ACN.
$HIF_{786-826}$ and $HIF_{776-826}$ were dissolved in H_2O and purified on a Phenomenex Luna C18 (250 x 10 mm) column using the following gradients: 5 min 0% B, 15 min 20% B, 75 min 50% B, 4 ml/min flow rate; or 5 min 0% B, 15 min 35% B, 75 min 65% B, 4 ml/min flow rate. Purity of the fractions were analysed by HPLC-UV using a Kinetex EVO C18 (5 mm, 100 Å, 250 x 4.6 mm) column and the following gradient: 0 min 5% B, 25 min 80% B, 1 ml/min flow rate.
$CITED_{216}$ and $CITED_{226}$ were dissolved in DMSO and purified on a Phenomenex Luna C4 (250 x 10 mm) column using the following gradient: 0 min 25% B, 5 min 25% B, 15 min 45% B, 70 min 75 %B, 4 ml/min. Fractions containing the peptides were collected, freeze-dried and repurified on the same column or on Kinetex EVO C18 (250 x 10 mm, 100 Å, 5 mm) using the following gradient: 0 min 25% B, 5 min 25% B, 15 min 50% B, 70 min 80 %B, 3 ml/min. Purity of the fractions were analysed using HPLC-UV on an Aeris Widepore C4 (250 x 4.6 mm) column using the following gradient: 0 min 10% B, 25 min 90 % B.

CD measurements

Measurements were performed on a Jasco J-1100 CD-spectrometer using a 1 mm cylindrical quartz cuvette at room temperature. Spectra were recorded from 195 to 250 nm at a scan speed of 100 nm min^{-1}, with 3 accumulations, which were averaged. Buffer baseline was subtracted. p300 was prepared in either 40 mM Na-phospate, 100 mM NaCl, 1 mM DTT, pH 7.4 buffer or dialysed into 25 mM Tris, 150 mM NaCl, 1 mM DTT pH 7.4 buffer.

Fluorescence anisotropy

Direct binding

GSTp300 protein was serially diluted in buffer (25 mM Tris-HCl, 150 mM NaCl, 1mM DTT, pH 7.4) and GFP-HIF-1α (50nM final concentration) was added, the plates were read after 0 min, 60 min, 4 h and 24 h, and incubated at room temperature in between. Each experiment was run in triplicate and the fluorescence anisotropy measured using an EnVision 2103 MultiLabel plate reader (Perkin Elmer) with excitation at 480 nm and emission at 535 nm (30 nm bandwidths). The recorded temperature for all measurements was 27°C ± 1°C. In parallel, a control experiment was performed in which no GFP-HIF was added and the volume made up with additional buffer, this blank was deducted from the raw data for each of the three repeats. The intensity was calculated for each point using eqn (1) and used to calculated anisotropy

using eqn (2). From a plot of anisotropy against GST-p300 concentration the minimum and maximum anisotropies were obtained using a logistic sigmoidal fit in OriginPro 8.6. This allowed the conversion to fraction bound (eqn (3)). The data were then fitted using eqn (4) in OriginPro 8.6 to determine the dissociation constant, Kd.

$$I = (2*PG) + S \qquad \text{(eq. 1)}$$

$$r = (S - PG)/I \qquad \text{(eq. 2)}$$

$$L_b = (r - r_{min})/((\lambda(r_{max} - r)) + r - r_{min}) \qquad \text{(eq. 3)}$$

$$y = ((K_d + x + [FL]) - \sqrt{((K_d + x + [FL])^2\ 4x[FL]))}/2 \qquad \text{(eq. 4)}$$

r = anisotropy, I = total intensity, P = perpendicular intensity, S = parallel intensity, G = an instrument factor set to 1, L_b = fraction ligand bound, $\lambda = I_{bound}/I_{unbound} = 1$, [FL] = concentration of fluorescent peptide, K_d = dissociation constant, y = L_b multiplied by [FL], x = protein concentration.

Isothermal titration calorimetry

Prior to ITC measurement p300 CH1 was dialysed or run on S75 column in the buffer used for titration, and the same buffer was used to dissolve the pure peptides. Experiments with expressed HIF-1α, CITED, CITIF and GFP-HIF-1α variant peptides were performed using a MicroCal iTC200 instrument with 10 µM protein in the cell and 100 µM ligand in the syringe at 37°C, using 2 µl injections and 120 or 180 s spacing between injections. Experiments with the synthetic HIF-1α, CITED and CITIF peptides were carried out using a Microcal VP ITC instrument using 3-5 µM protein in the cell and 40-60 µM ligand in the syringe at 35°C, using 5 µl injections and 180 s spacing between injections, for 40 injections. Titrations were performed in 40 mM Na-phosphate, pH 7.5 100 mM NaCl, 1 mM DTT buffer except for GFP-HIF-1α and variants for which 25 mM Tris-HCl, 150 mM NaCl, 1mM DTT, pH 7.4 buffer was used. Data were analysed using NITPIC[3] and fitted using SEDPHAT[4] to a single binding site model (fixed 1:1 stoichiometry), with baseline and incompetent protein fraction as adjustable parameters. 68 % confidence intervals for the fitted values were determined using the automatic confidence interval search implemented in SEDPHAT. Figures were prepared using GUSSI[5].

Competition ITC measurements

Competition ITC measurements were performed in Microcal VP ITC. For competition ITC measurements the P300/ligand complexes were prepared by titrating the appropriate ligand first to p300 in the cell using 3-5 µM protein concentration, the titration stopped when it reached close to saturation conditions (i.e. no significant difference in injection heats). This resulted in

1.15-1.5 excess of ligand compared to p300 in the cell, the recalculated concentrations for each experiment are listed in Table S4. The additional volume was removed from the cell and the complex was titrated with the competitor ligand using 20-50 μM concentration in the syringe, 5 or 10 uL injections with 180-240 s spacing. Background titrations were performed without p300 in the cell and subtracted from the measurements. Integration was performed using NITPIC and data were fitted using SEDPHAT using two different models described below.

Table S5 Concentrations used in competition ITC experiments.

	Cell	*Syringe*	*Injection volume / spacing*
$CITED2_{224\text{-}259}$ titrated to HIF-1α$_{786\text{-}826}$/P300 complex	4.5 μM P300<br>5.5 μM $HIF_{786\text{-}826}$	50 μM $CITED_{224\text{-}259}$	8 μl / 240 s
$CITED2_{224\text{-}259}$ titrated to HIF-1α$_{776\text{-}826}$/P300 complex	4.5 μM P300<br>6.7 μM $HIF_{776\text{-}826}$	60 μM $CITED_{224\text{-}259}$	10 μl / 240 s
HIF-1α $_{786\text{-}826}$ titrated to $CITED2_{224\text{-}259}$ /P300 complex	4.4 μM P300<br>5.9 μM $CITED_{224\text{-}259}$	50 μM $HIF_{786\text{-}826}$	10 μl / 240 s
$CITED2_{216\text{-}269}$ titrated to HIF-1α $_{776\text{-}826}$/P300 complex	2.1 μM P300<br>3.6 μM $HIF_{776\text{-}826}$	20 μM $CITED_{216\text{-}269}$	10 μl / 240 s
HIF-1α $_{776\text{-}826}$ titrated to $CITED2_{216\text{-}259}$ /P300 complex	5 μM P300<br>5.8 μM $CITED_{216\text{-}269}$	50 μM $HIF_{776\text{-}826}$	5 μl / 180 s
$CITED2_{216\text{-}248}$ titrated to HIF-1α $_{776\text{-}826}$/P300 complex	4.4 μM P300<br>5.9 μM $HIF_{776\text{-}826}$	50 μM $CITED_{216\text{-}248}$	10 μl / 240 s

Fitting model 1.: Data analysis using competition model

In order to obtain apparent binding parameters, the following competition model was used (in SEDPHAT "A+B+C <-> AB + C <-> AC + B, competing B and C for A" model) :

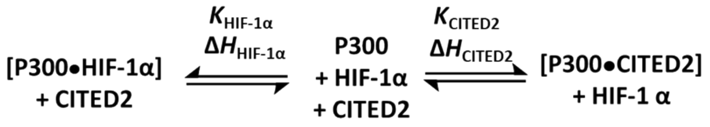

Figure S17 Parametrized arrow diagram of the competition model

The fitted parameters were $K_{HIF-1α}$, K_{CITED2}, $ΔH_{HIF-1α}$, $ΔH_{CITED2.}$ including an incompetent fraction fit for protein and baseline correction. For titrations which were performed by titrating CITED2 into the preformed complex of HIF-1α/p300 the parameters $K_{HIF-1α}$ and $ΔH_{HIF-1α}$ were kept constant using the previously determined values. For titrations which were performed by titrating HIF-1α into the preformed complex of CITED2/p300 the parameters K_{CITED2} and $ΔH_{CITED2}$ were kept constant using the previously determined parameters. Detailed fitting equations can be found in ref 4 and 6.

Fitting model 2.: Global analysis including ternary complex formation

Global analyses[7,8] of the competition experiments were performed using the model shown in Figure S18.

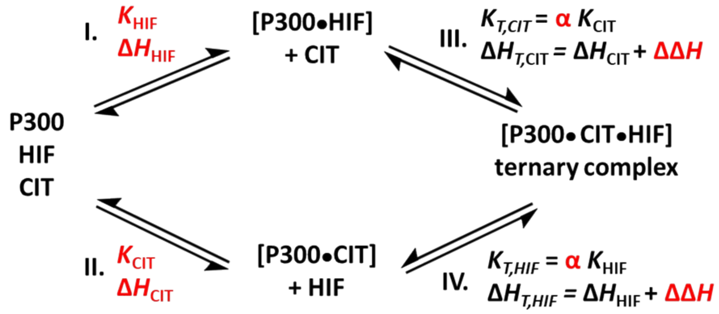

Figure S18 Arrow diagram of the model used to fit cooperative parameters for the CITED2, HIF-1α, p300 system. Fitted parameters are indicated in red, sequence numbers are not shown for clarity. K_{HIF} ($ΔH_{HIF}$) and K_{CIT} ($ΔH_{CIT}$) are the association constant (and enthalpy change) for HIF-1α or CITED2 binding to p300 (Equilibra I. and II.) $K_{T,CIT}$ ($ΔH_{T,CIT}$) and $K_{T,HIF}$ ($ΔH_{T,HIF}$) are the association constant (and enthalpy change) for CITED2 binding to a preformed HIF-1α/p300 complex (equilibrium III.) or for HIF-1α binding to a preformed p300/CITED2 complex (Equilibrium IV.) α is the cooperativity constant (α =0 competitive, α >1 positive and α <1 negative cooperativity) and ΔΔH is the cooperativity enthalpy (Eq. 16).

In this fitting model it is assumed that due to microscopic reversibility the cooperativity of adding CITED2 to a preformed HIF-1α /p300 complex is equal to the cooperativity of adding HIF-1α to the pre-existing CITED2/p300 complex (i.e the presence of CITED2 modifies the affinity of HIF-1α in the same way as the presence of HIF-1α modifies the affinity of CITED2):

$$[p300\iota HIF] = K_{HIF}[p300][HIF] \quad Eq.5.$$

$$[p300\iota CIT] = K_{CIT}[p300][CIT] \quad Eq.6.$$

$$[p300\iota CIT\iota HIF] = K_{T,CIT}[p300\iota HIF][CIT] \quad Eq.7.$$

$$[p300\iota CIT\iota HIF] = K_{T,HIF}[p300\iota CIT][HIF] \quad Eq.8.$$

When each of the reactions are considered separately at equilibrium, the association constants are dependent upon each other:

$$K_{HIF}K_{T,CIT} = K_{CIT}\,K_{T,HIF} \quad Eq.9.$$

introducing an interaction cooperativity constant α, which determines whether the ternary complex formation is favourable or unfavourable:

$$K_{T,CIT} = \alpha *\ K_{CIT} \quad Eq.10.$$

$$K_{T,HIF} = \alpha * K_{HIF} \quad Eq.11.$$

The Gibbs energy for the formation of each complex:

$$\Delta G_{HIF} = \ -RTlnK_{HIF} \quad Eq.12.$$

$$\Delta G_{CIT} = \ -RTlnK_{CIT} \quad Eq.13.$$

$$\Delta G_{T} = \ -RTln(\alpha K_{HIF}K_{CIT}) = \ \Delta G_{HIF} + \ \Delta G_{CIT} + \ \Delta\Delta G \quad Eq.14.$$

where ΔG_T is the Gibbs energy associated with the formation of the ternary complex. α is a temperature dependent equilibrium constant and related to the cooperativity Gibbs energy, entalpy and entropy (by applying the Gibbs-Helmholtz relationship):

$$\Delta\Delta G = \ -RTln\alpha = \ \Delta G_{T} - \ \Delta G_{CIT} - \ \Delta G_{HIF} \quad Eq.15.$$

$$\Delta\Delta H = \ RT^2\left(\frac{\partial ln\alpha}{\partial T}\right)_P = \ \Delta H_T - \Delta H_{HIF} - \ \Delta H_{CIT} \quad Eq.16.$$

$$\Delta\Delta S = \ R\left(ln\alpha + T\left(\frac{\partial ln\alpha}{\partial T}\right)_P\right) = \ \Delta S_T - \Delta S_{HIF} - \ \Delta S_{CIT} \quad Eq.17.$$

The enthalpy associated with the ternary complex formation (ΔH_T) can be given by:

$$\Delta H_T = \ \Delta H_{HIF} + \Delta H_{CIT} + \Delta\Delta H \quad Eq.18.$$

Thus the enthalpy change for equilibrium III. (CITED2 binding to HIF-1 α /p300 complex can be given by:

$$\Delta H_{T,CIT} = \Delta H_{CIT} + \Delta\Delta H \qquad Eq. 19.$$

And the enthalpy for equilibrium IV. HIF-1 α binding to CITED2/p300 complex can be given by:

$$\Delta H_{T,HIF} = \Delta H_{HIF} + \Delta\Delta H \qquad Eq. 20.$$

The equations are derived from the analysis used in ref 8 and 9.

Fitting in SEDPHAT was parametrized as follows: HIF-1$\alpha_{776\text{-}826}$ titrated to p300 (B to A); CITED2$_{216\text{-}269}$ or CITED2$_{224\text{-}259}$ or CITED2$_{216\text{-}248}$ titrated to p300 (C to A); CITED2$_{216\text{-}269}$ or CITED2$_{224\text{-}259}$ or CITED2$_{216\text{-}248}$ titrated to p300/HIF-1$\alpha_{776\text{-}826}$ complex (C to AB). K_A, ΔH_A, K_B, ΔH_B, $\log K_{[AB]C}/K_{AC}$ and $\Delta\Delta H$ (corresponding to K_{HIF}, ΔH_{HIF}, K_{CITED}, ΔH_{CITED}, cooperativity constant α and $\Delta\Delta H$) were fitted using A+B+C <-> AB + C <-> AC + B <-> ABC triple complex model. Incompetent fraction for p300 and baseline was fitted locally for each experiment. 68 % confidence intervals for the fitted values were determined using the automatic confidence interval search implemented in SEDPHAT.

Co-crystallization

p300 was mixed with CITIF and the complex was purified by size-exclusion chromatography (S75 26/600 pg column in 20 mM Tris, 150 mM NaCl, 1mM DTT pH 7.5 buffer). Fractions containing the complex were concentrated to a final protein concentration of 5-6 mg/ml. Sparse matrix screening using the JCSG Core suites (Qiagen) was performed with the sitting-drop vapor-diffusion method at 20 °C. Protein was mixed with crystallization solution at a 1:1 ratio with final drop volume of 0.2 µl using NT8® crystallization robot and the Rock Maker® platform (Formulatrix). Initial hit conditions were further optimized by screening HEPES pH 6 – 7.5 in 0.5 pH steps and PEG 6K concentration from 15-40% in 5% steps. Crystals were flash cooled in liquid nitrogen and sent to Diamond Light Source (DLS) for data collection. Data were collected at 100 K. Data were processed with the xia2[10] bundle using DIALS[11] for integration and Pointless[12], Aimless[13] for scaling and merging. Phasing was performed by molecular replacement using Phaser[14] and using 7LVS.pdb as the search model. Initial refinement was done using REFMAC[15] with model building in COOT[16] using the CCP4i2[17] software package. Further refinements and TLS refinement were done in PHENIX[18] and the structures were analysed by Molprobity[19].

Table S6 Oligonucleotide sequences used for site-directed mutagenesis

L795A	5'-acttcacaatcataactggtcgcctgtggtaatccactttcatc-3'
	5'-gatgaaagtggattaccacaggcgaccagttatgattgtgaagt-3'
D799A	5'-ggagcattaacttcacaagcataactggtcagctgtg-3'
	5'-cacagctgaccagttatgcttgtgaagttaatgctcc-3'
E801A	5'-gctgaccagttatgattgtgcagttaatgctcctatacaag-3'
	5'-cttgtataggagcattaactgcacaatcataactggtcagc-3'
N803A	5'-ccttgtataggagcagcaacttcacaatcataactggtcagctgtg-3'
	5'-cacagctgaccagttatgattgtgaagttgctgctcctatacaagg-3'
L818A	5'-tgatccaaagctctgagtgcttcttcaccctgcagtaggt-3'
	5'-acctactgcagggtgaagaagcactcagagctttggatca-3'
L822A	5'-gagctagttaacttgatccgcagctctgagtaattcttcaccc-3'
	5'-gggtgaagaattactcagagctgcggatcaagttaactagctc-3'
D823A	5'-ctcgagctagttaacttgagccaaagctctgagtaattc-3'
	5'-gaattactcagagctttggctcaagttaactagctcgag-3'
V825A	5'-ggtgctcgagctagttagcttgatccaaagctctg-3'
	5'-cagagctttggatcaagctaactagctcgagcacc-3'
L792A	5'-ctggtcagctgtggtgctccactttcatccattgattgcc-3'
	5'-ggcaatcaatggatgaaagtggagcaccacagctgaccag-3'
E817A	5'-ccaaagctctgagtaatgcttcaccctgcagtagg-3'
	5'-cctactgcagggtgaagcattactcagagctttgg-3'
S797A	5'-gcattaacttcacaatcataagcggtcagctgtggtaatccac-3'
	5'-gtggattaccacagctgaccgcttatgattgtgaagttaatgc-3'
Q824A	5'-gtgctcgagctagttaactgcatccaaagctctgagtaattcttc-3'
	5'-gaagaattactcagagctttggatgcagttaactagctcgagcac-3'
L818A, L822A	5'-gagctagttaacttgatccgcagctctgagtgcttcttcaccctgcagtaggttt-3'
	5'-aaacctactgcagggtgaagaagcactcagagctgcggatcaagttaactagctc-3'
L818A L822A V825A	5'-ggtgctcgagctagttagcttgatccgcagctctgagtgcttcttcaccctgcagta-3'
	5'-tactgcagggtgaagaagcactcagagctgcggatcaagctaactagctcgagcacc-3'
L818A L822A D823A	5'-gaaacctactgcagggtgaagaagcactcagagctgcggctcaagttaactagctcgagc-3'
	5'-gctcgagctagttaacttgagccgcagctctgagtgcttcttcaccctgcagtaggtttc-3'
L795A D799A	5'-tataggagcattaacttcacaagcataactggtcgcctgtggtaatccactttcatcc-3'
	5'-ggatgaaagtggattaccacaggcgaccagttatgcttgtgaagttaatgctcctata-3'
L795A D799A E801A	5'-ccttgtataggagcattaactgcacaagcataactggtcgcctgtggtaatccactttcatc-3'
	5'-gatgaaagtggattaccacaggcgaccagttatgcttgtgcagttaatgctcctatacaagg-3'
L795A D799A E801A N803A	5'-tctgctgccttgtataggagcagcaactgcacaagcataactggtcgcctgtggtaatccactttcatcc-3'
	5'-ggatgaaagtggattaccacaggcgaccagttatgcttgtgcagttgctgctcctatacaaggcagcaga-3'

Peptide characterization data

HIF-1α$_{786\text{-}826}$ (Synthetic)

Ac-SMDESGLPQLTSYDCEVNAPIQGSRNLLQGEELLRALDQVN-NH_2

Em: 4543.185, MW: 4546.001

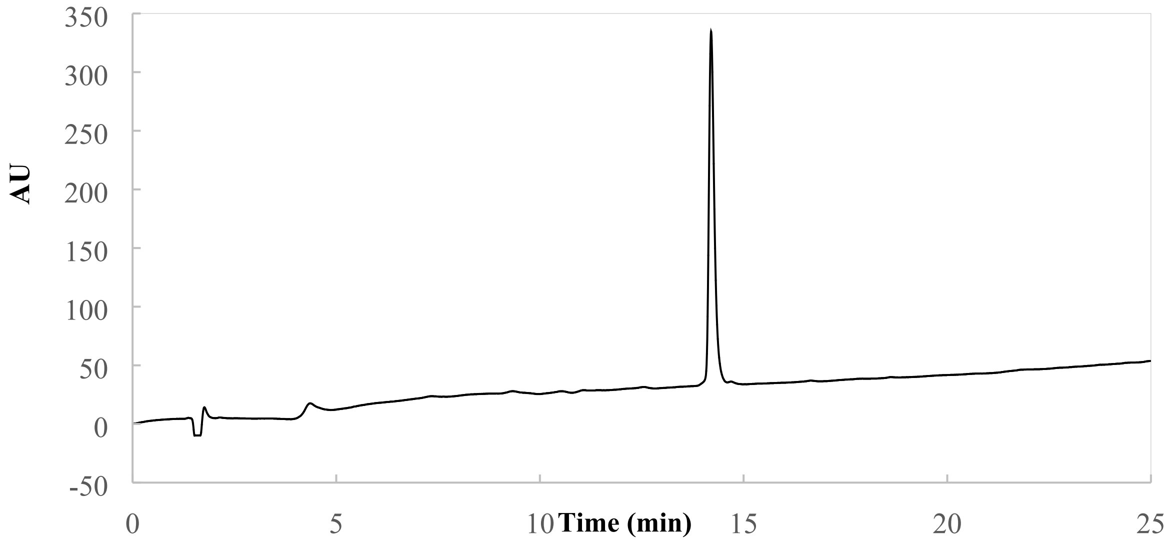

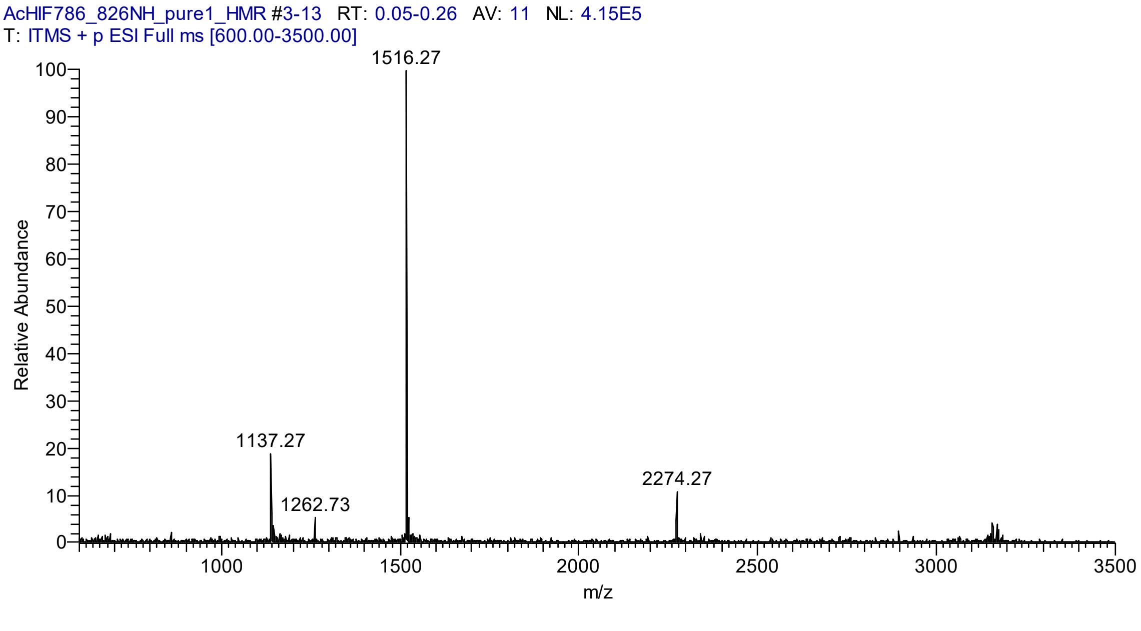

HIF-1α$_{776-826}$ (Synthetic)

Ac-SDLACRLLGQSMDESGLPQLTSYDCEVNAPIQGSRNLLQGEELLRALDQVN-NH_2

Em: 5599.724;

Mw: 5603.236

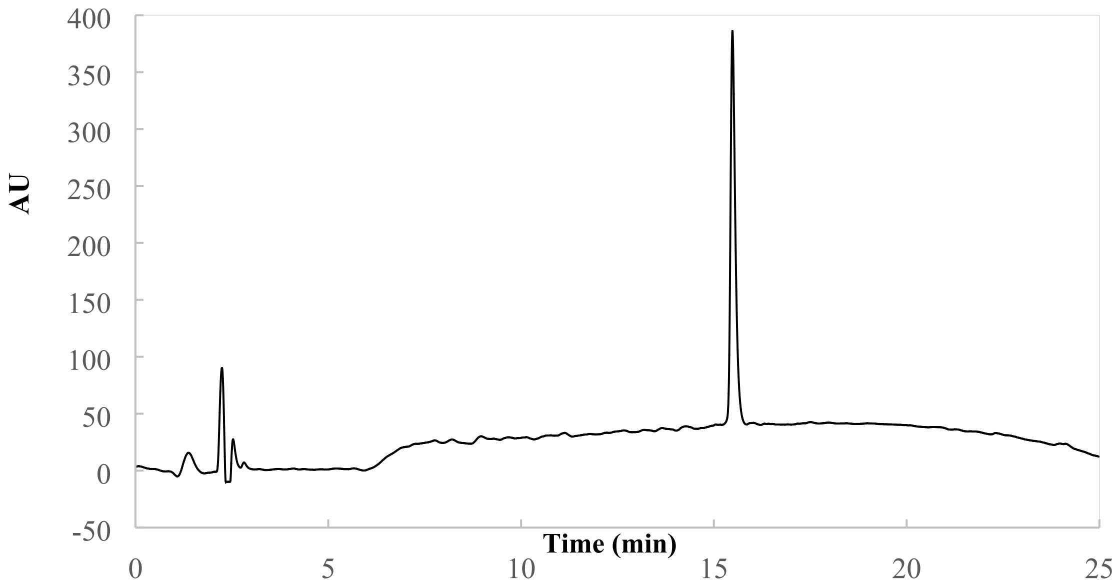

98s

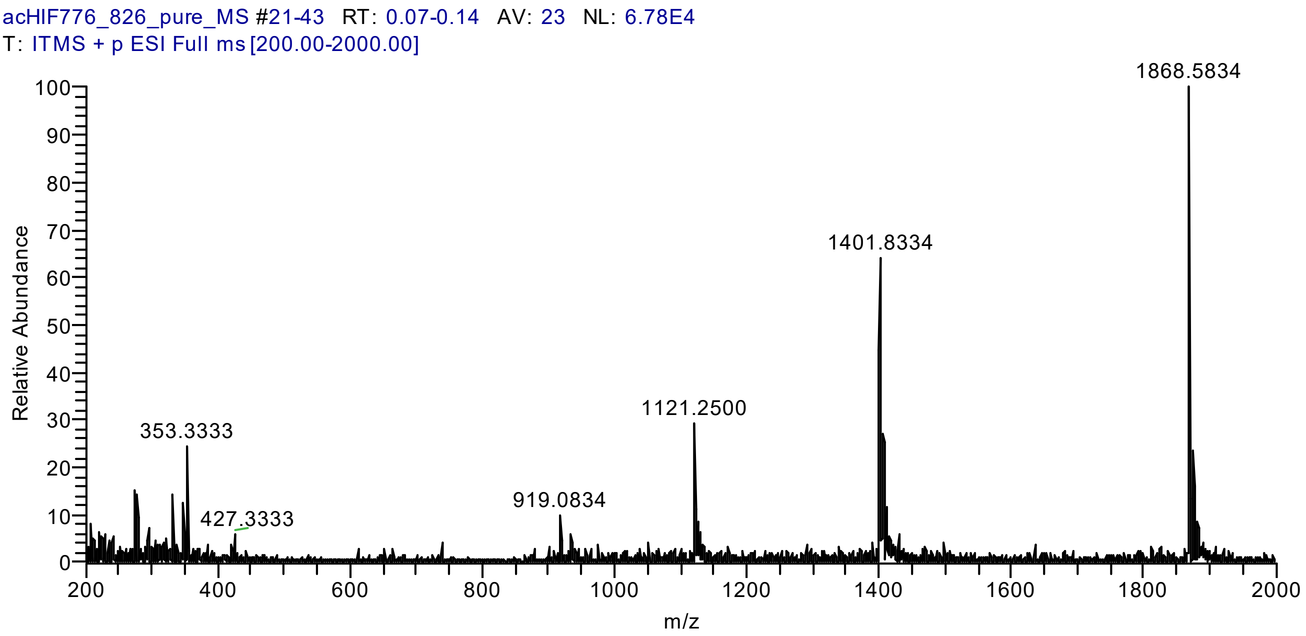

CITED2$_{224-259}$ (Synthetic)

Ac-DEEVLMSLVIEMGLDRIKELPELWLGQNEFDFMTDF-NH_2

Em: 4342.078

Mw: 4344.963

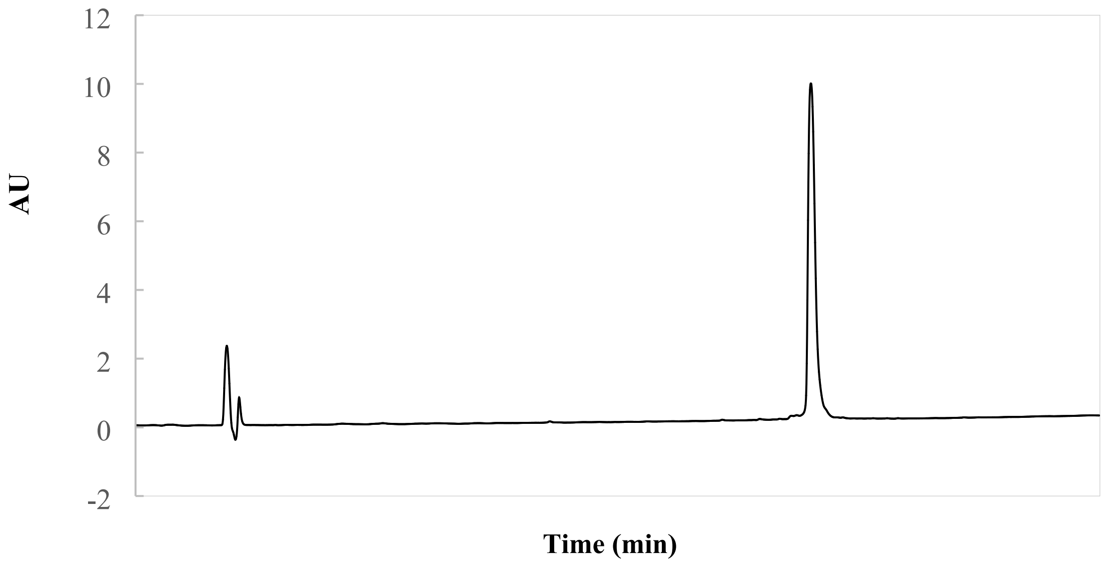

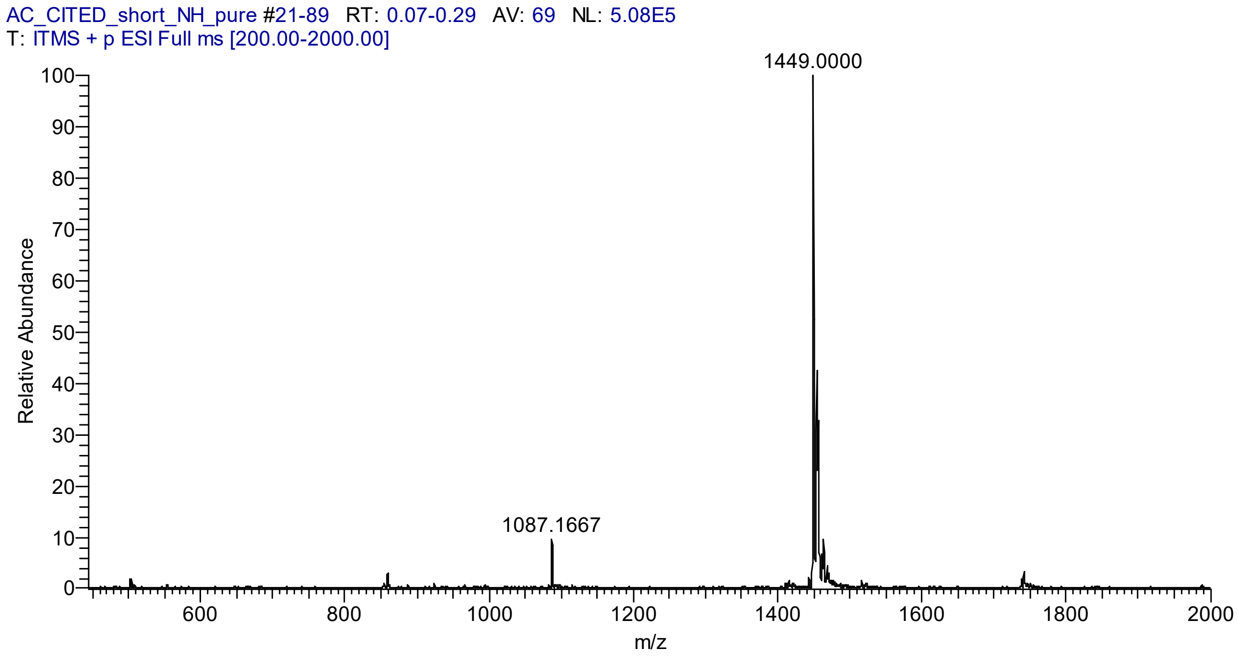

CITED2$_{216\text{-}269}$ (Synthetic)

Ac-NVIDTDFIDEEVLMSLVIEMGLDRIKELPELWLGQNEFDFMTDFVCKQQPSRVS-NH_2

Em: 6372.104

MW: 6376.276

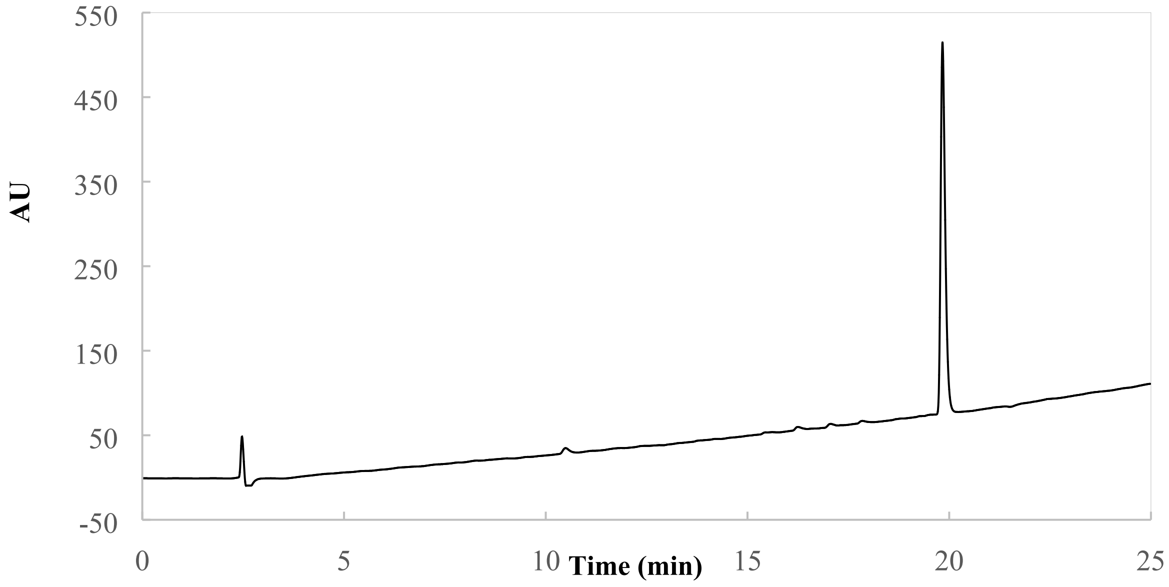

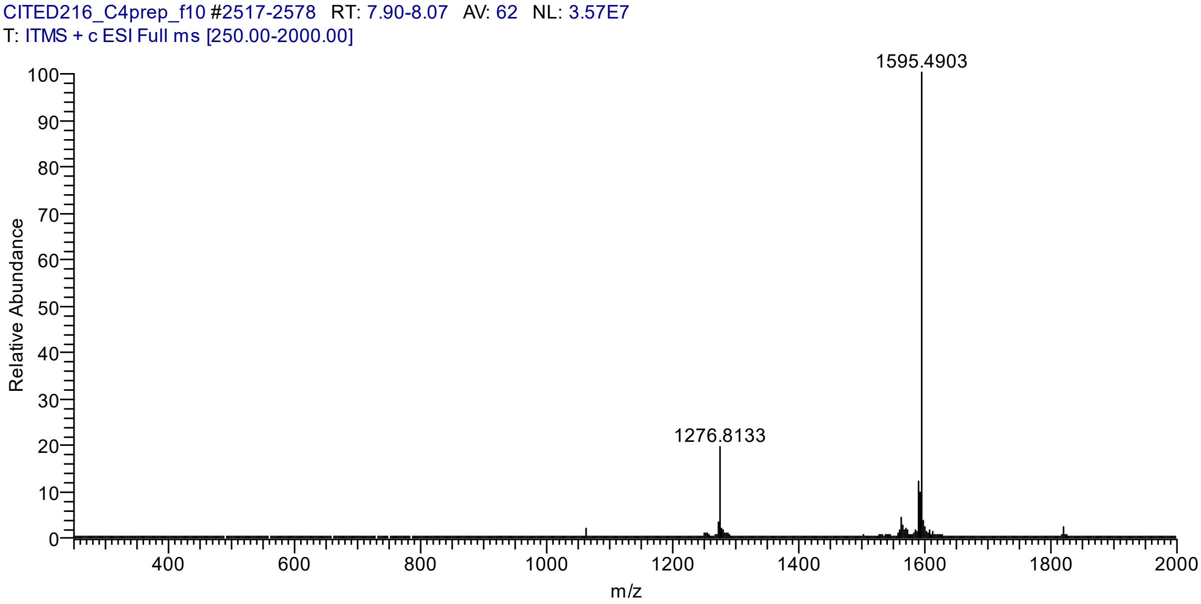

CITIF (synthetic)

Ac-DEEVLMSLVIEMGLDRIKELPQLTSYDCEVNAPIQGSRNLLQGEELLRALDQVN-NH_2

Em: 6137.102

Mw: 6140.975

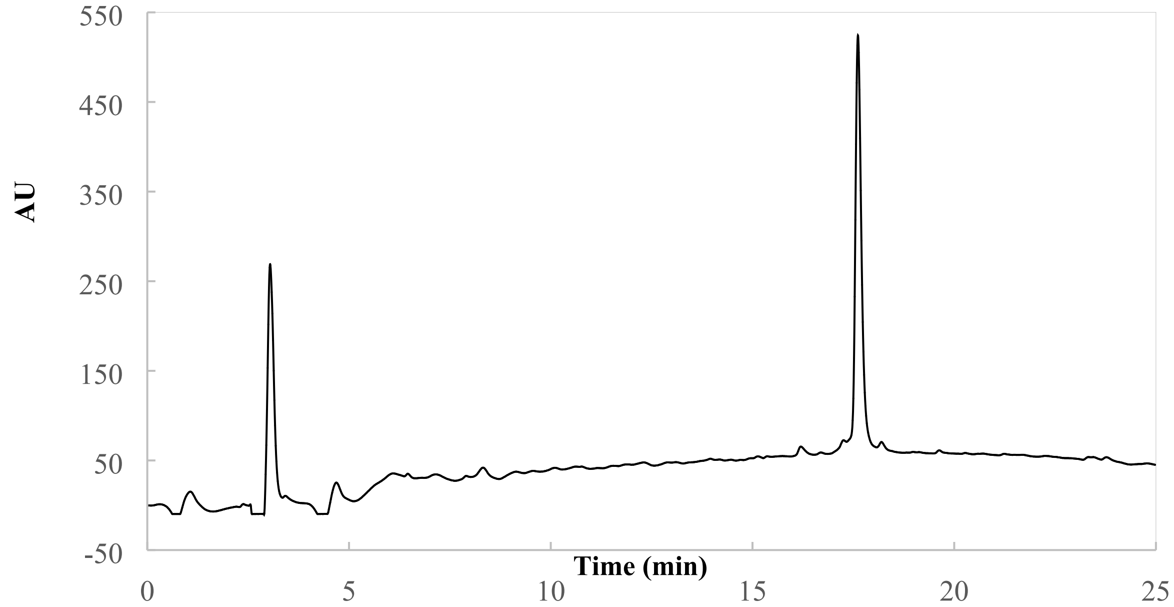

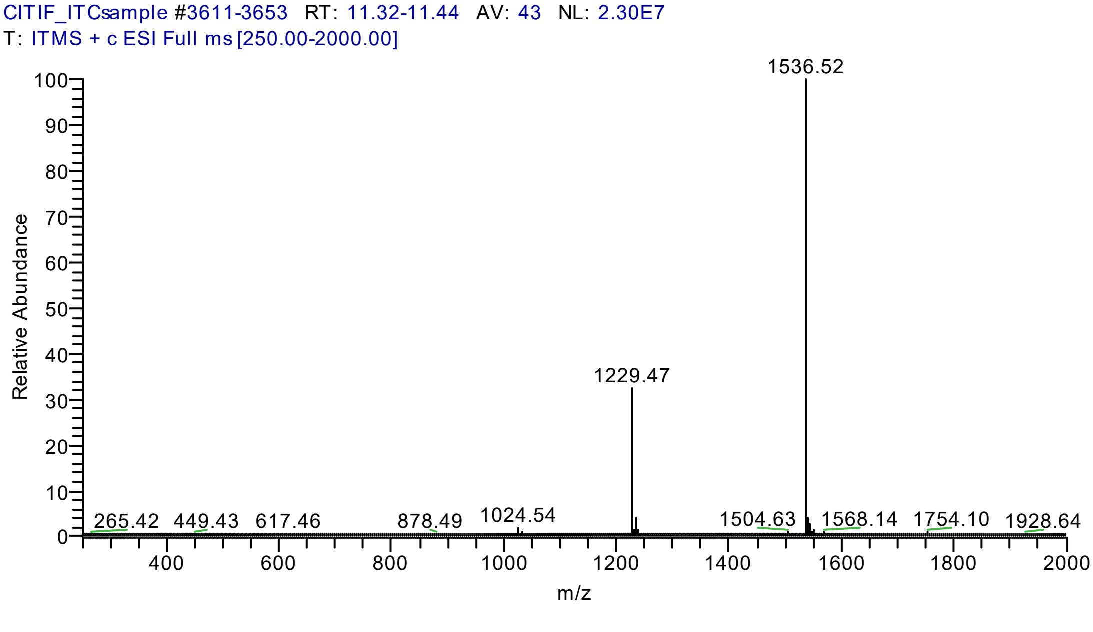

CITED2$_{216-248}$

Ac-NVIDTDFIDEEVLMSLVIEMGLDRIKELPELW-NH_2

Em: 3814,93

Mw: 3817,41

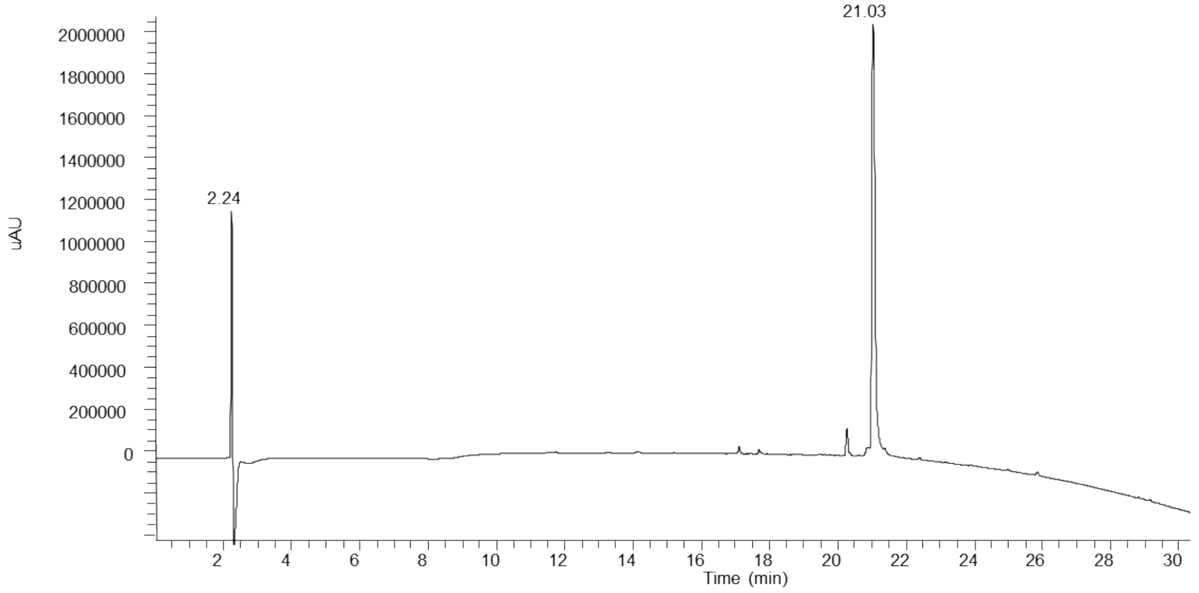

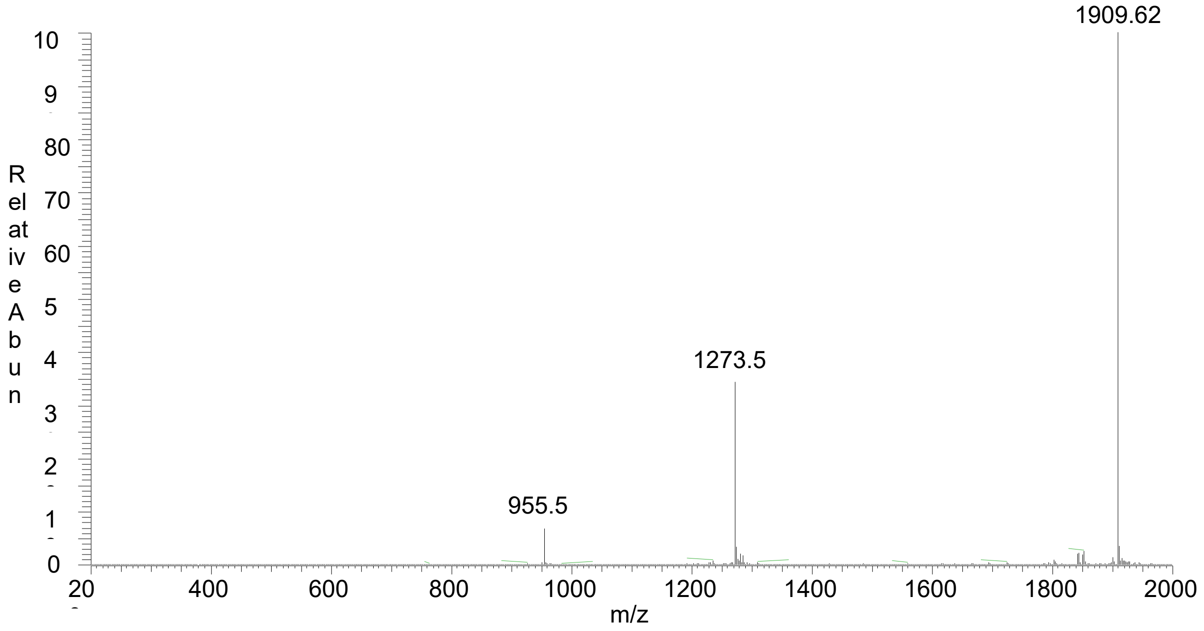

CITED2$_{224-248}$

Ac-DEEVLMSLVIEMGLDRIKELPELW-NH_2

Em: 2897,48

Mw: 2899,40

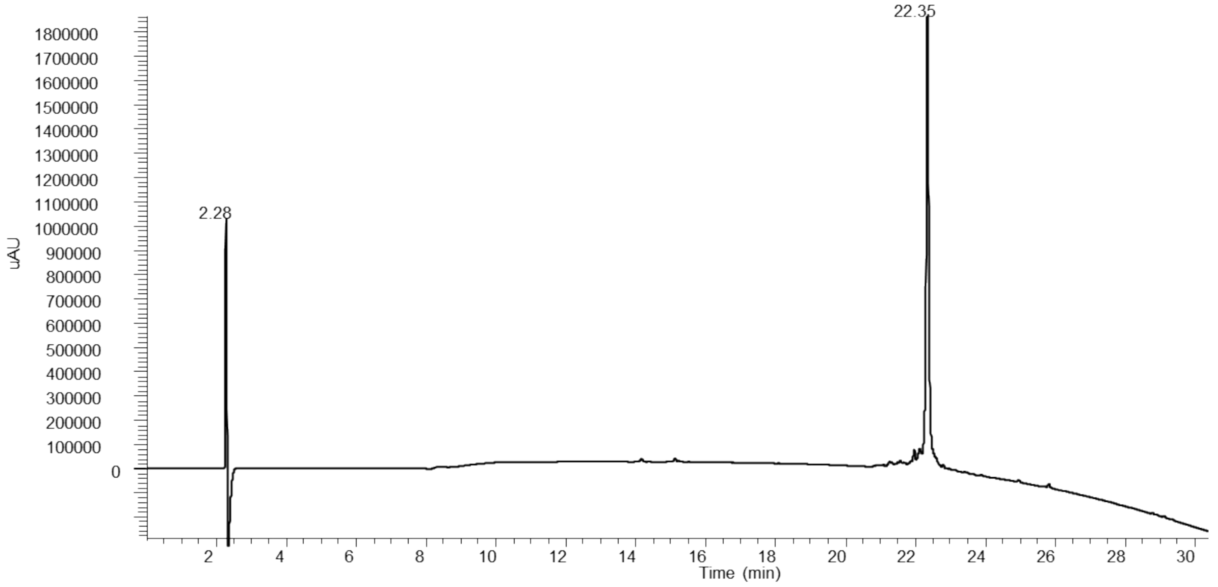

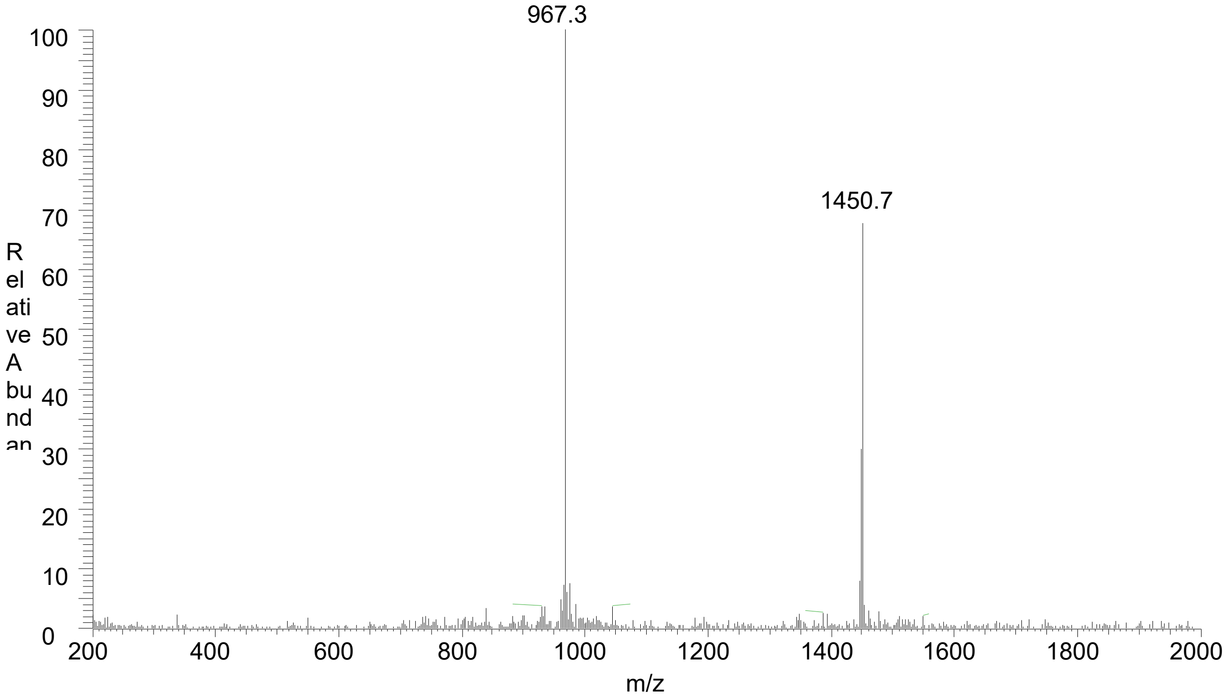

Characterization data for the expressed HIF-1α and CITED and CITIF constructs

HIF-1α$_{776\text{-}826}$

GPGSSDLACRLLGQSMDESGLPQLTSYDCEVNAPIQGSRNLLQGEELLRALDQVN-OH

Em: 5856.825

MW: 5860.481

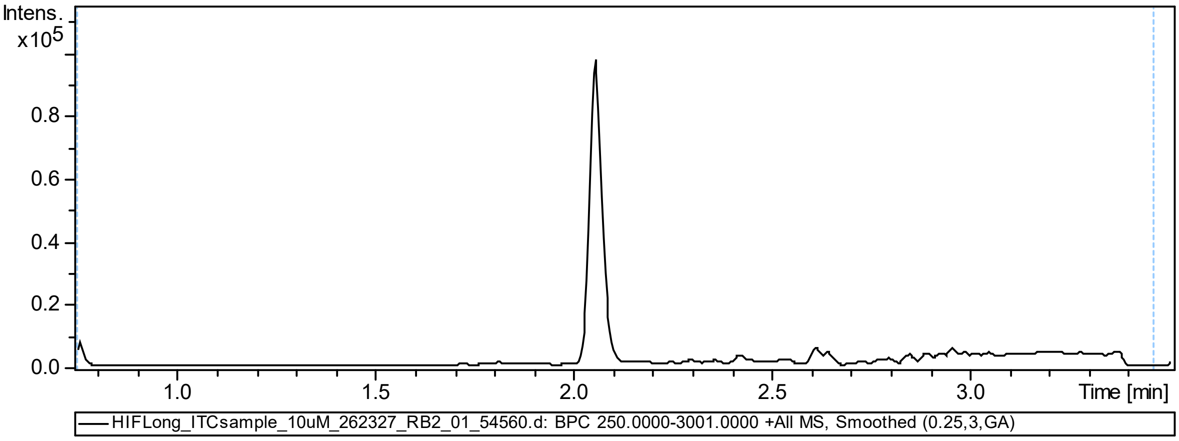

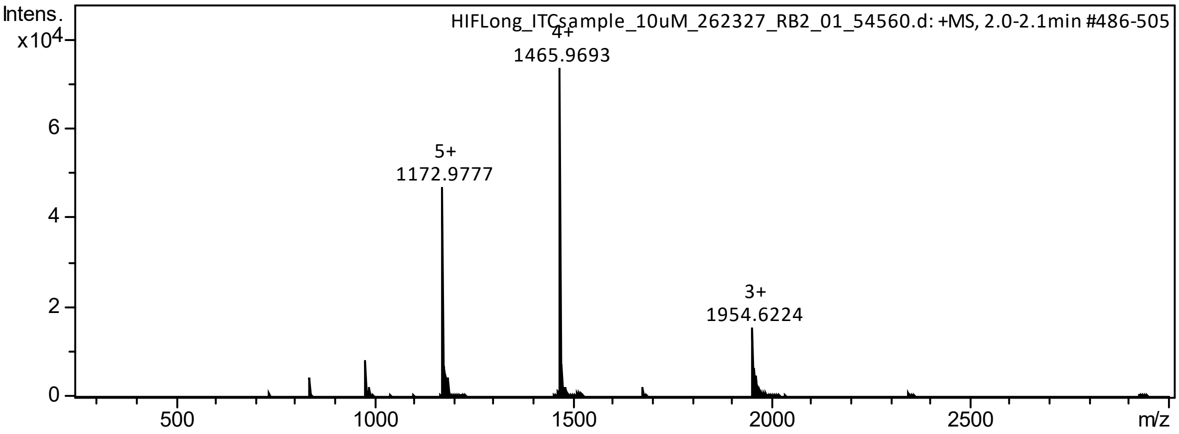

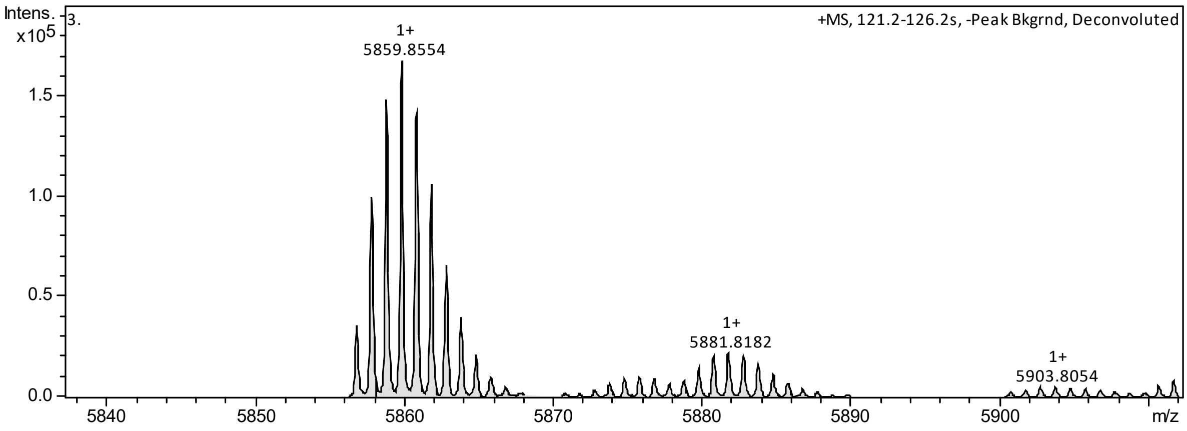

CITED2$_{224\text{-}259}$ (expressed)

GPGSDEEVLMSLVIEMGLDRIKELPELWLGQNEFDFMTDF-OH

MW: 4602.208

Em: 4599.179

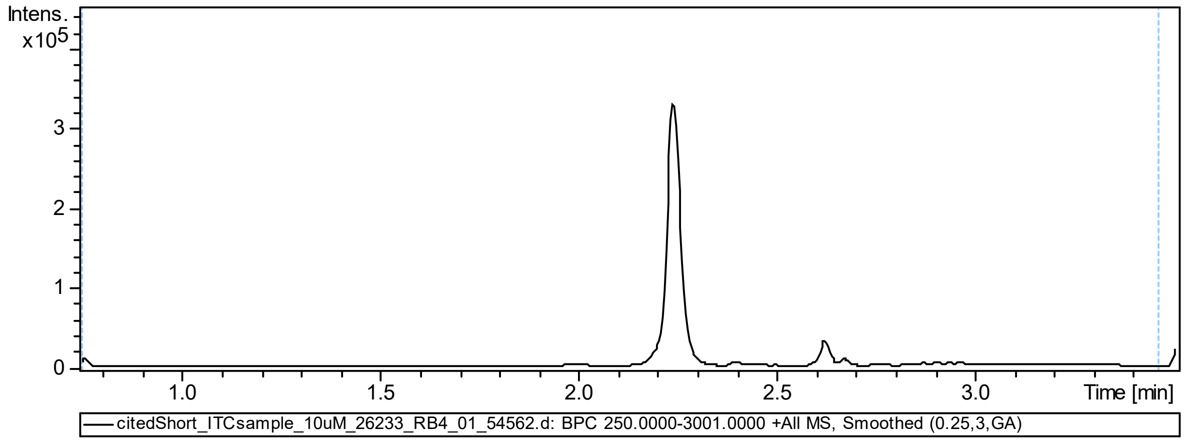

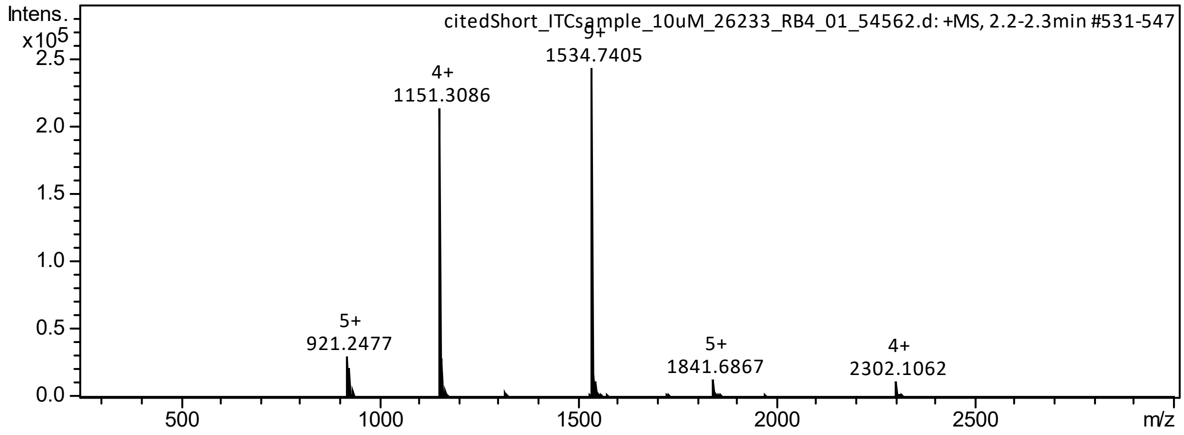

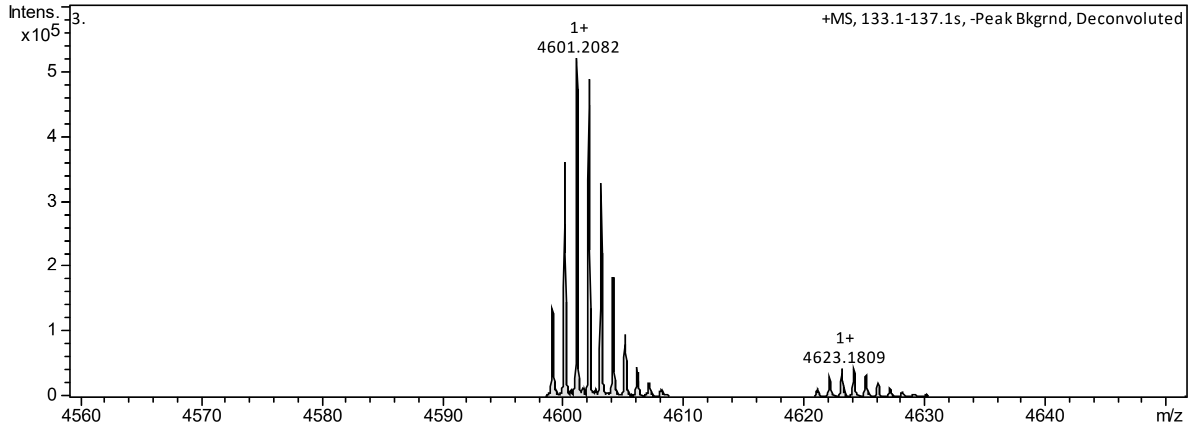

CITED2$_{216-269}$ (expressed)

GPGSNVIDTDFIDEEVLMSLVIEMGLDRIKELPELWLGQNEFDFMTDFVCKQQPSRVS-OH

MW: 6633.521

Em:6629.205

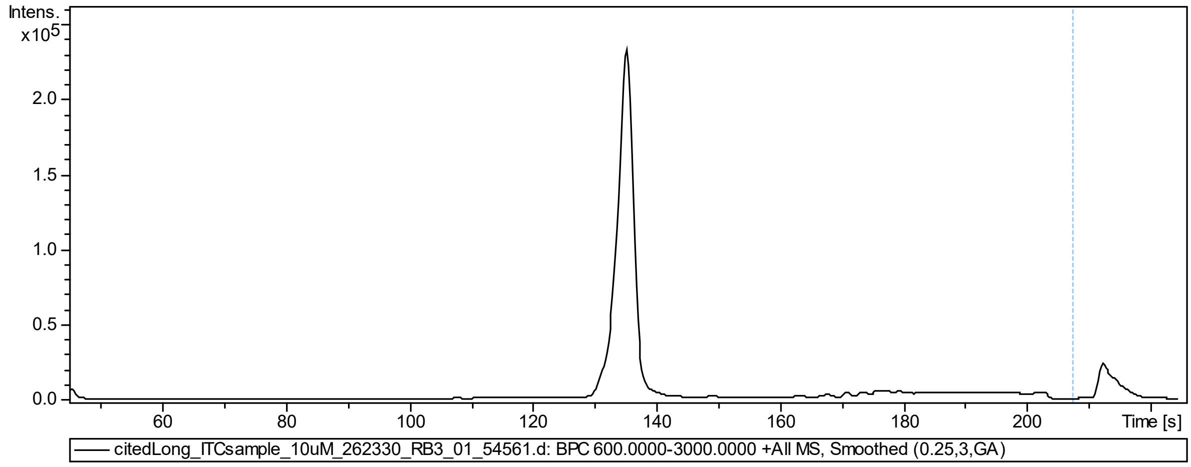

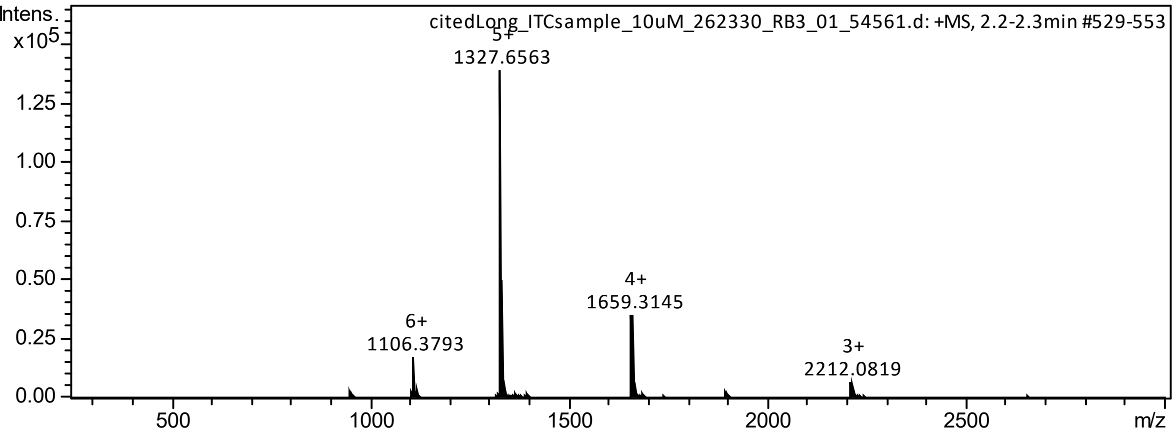

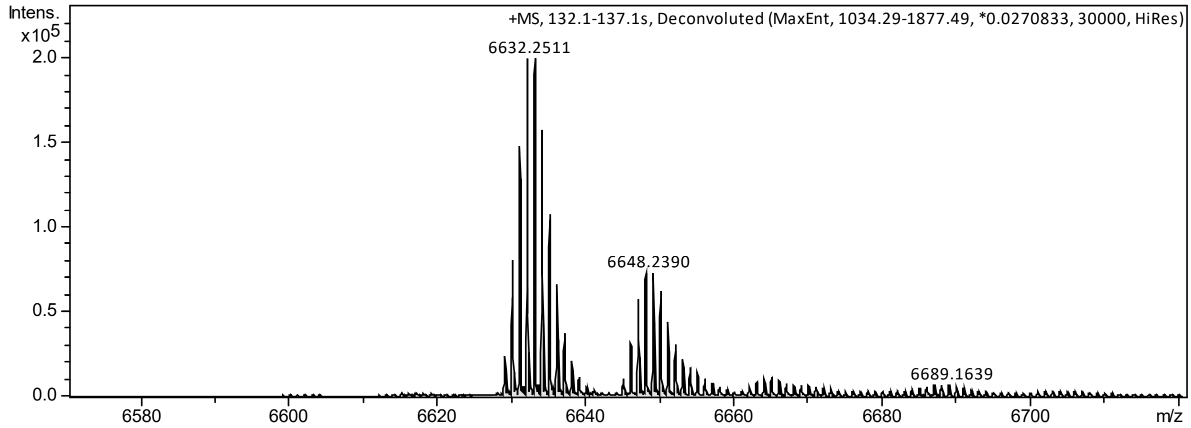

CITIF (expressed)

GPGSDEEVLMSLVIEMGLDRIKELPQLTSYDCEVNAPIQGSRNLLQGEELLRALDQVN-OH

MW: 6398.220

Em: 6394.203

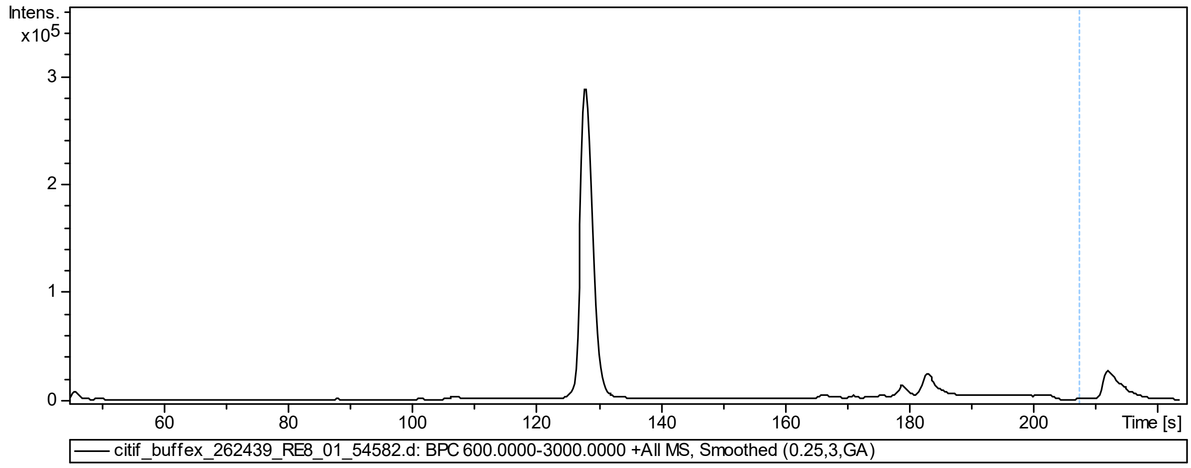

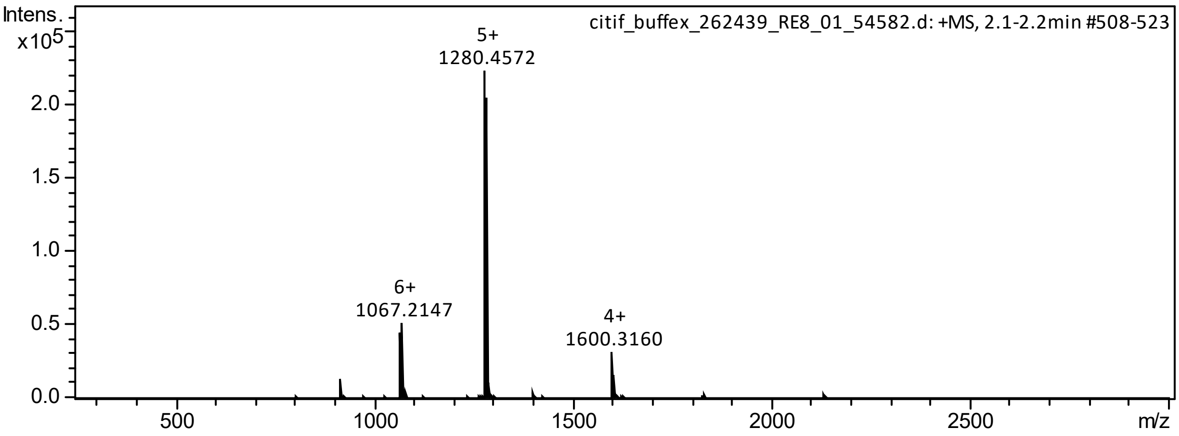

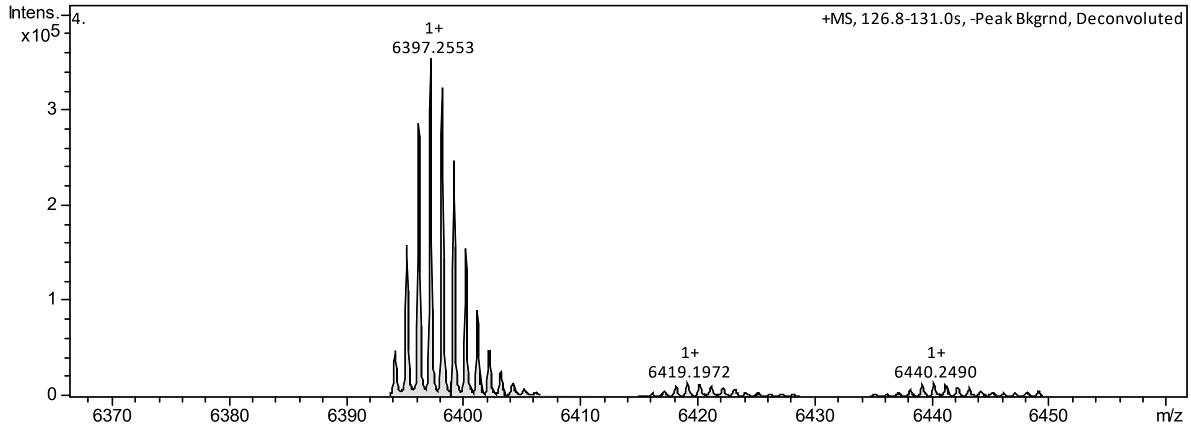

References

(1) Wood, C. W.; Ibarra, A. A.; Bartlett, G. J.; Wilson, A. J.; Woolfson, D. N.; Sessions, R. B. BAlaS: Fast, Interactive and Accessible Computational Alanine-Scanning Using BudeAlaScan. BIOINFORMATICS 36 (9), 2917–2919, (2020).

(2) Kyle, H. F.; Wickson, K. F.; Stott, J.; Burslem, G. M.; Breeze, A. L.; Tiede, C.; Tomlinson, D. C.; Warriner, S. L.; Nelson, A.; Wilson, A. J.; Edwards, T. A. Exploration of the HIF-1a/P300 Interface Using Peptide and Adhiron Phage Display Technologies †. 2738 | MOL. BIOSYST 11, 2738, (2015).

(3) Keller, S.; Vargas, C.; Zhao, H.; Piszczek, G.; Brautigam, C. A.; Schuck, P. High-Precision Isothermal Titration Calorimetry with Automated Peak-Shape Analysis. ANALYTICAL CHEMISTRY 84 (11), 5066–5073, (2012).

(4) Zhao, H.; Piszczek, G.; Schuck, P. SEDPHAT – A Platform for Global ITC Analysis and Global Multi-Method Analysis of Molecular Interactions. METHODS 76, 137–148, (2015).

(5) Brautigam, C. A. Calculations and Publication-Quality Illustrations for Analytical Ultracentrifugation Data. METHODS IN ENZYMOLOGY 562, 109–133, (2015).

(6) Krainer, G.; Broecker, J.; Vargas, C.; Keller, S. Quantifying High-A Ffi Nity Binding of Hydrophobic Ligands by Isothermal Titration Calorimetry. (2012).

(7) Brautigam, C. A.; Zhao, H.; Vargas, C.; Keller, S.; Schuck, P. Integration and Global Analysis of Isothermal Titration Calorimetry Data for Studying Macromolecular Interactions. NATURE PROTOCOLS 2016 11:5 11 (5), 882–894, (2016).

(8) Houtman, J. C. D.; Brown, P. H.; Bowden, B.; Yamaguchi, H.; Appella, E.; Samelson, L. E.; Schuck, P. Studying Multisite Binary and Ternary Protein Interactions by Global Analysis of Isothermal Titration Calorimetry Data in SEDPHAT: Application to Adaptor Protein Complexes in Cell Signaling. PROTEIN SCIENCE : A PUBLICATION OF THE PROTEIN SOCIETY 16 (1), 30, (2007).

(9) Velazquez-Campoy, A.; Goñi, G.; Peregrina, J. R.; Medina, M. Exact Analysis of Heterotropic Interactions in Proteins: Characterization of Cooperative Ligand Binding by Isothermal Titration Calorimetry. BIOPHYSICAL JOURNAL 91 (5), 1887–1904, (2006).

(10) Winter, G. Xia2: An Expert System for Macromolecular Crystallography Data Reduction. JOURNAL OF APPLIED CRYSTALLOGRAPHY 43 (1), 186–190, (2010).

(11) Winter, G.; Waterman, D. G.; Parkhurst, J. M.; Brewster, A. S.; Gildea, R. J.; Gerstel, M.; Fuentes-Montero, L.; Vollmar, M.; Michels-Clark, T.; Young, I. D.; Sauter, N. K.; Evans, G. DIALS: Implementation and Evaluation of a New Integration Package. ACTA CRYSTALLOGRAPHICA SECTION D: STRUCTURAL BIOLOGY 74 (2), 85–97, (2018).

(12) Evans, P. Scaling and Assessment of Data Quality. ACTA CRYSTALLOGRAPHICA SECTION D: BIOLOGICAL CRYSTALLOGRAPHY 62 (1), 72–82, (2006).

(13) Evans, P. R.; Murshudov, G. N. How Good Are My Data and What Is the Resolution? Acta Crystallographica Section D: Biological Crystallography 69 (7), 1204–1214, (2013).

(14) McCoy, A. J.; Grosse-Kunstleve, R. W.; Adams, P. D.; Winn, M. D.; Storoni, L. C.; Read, R. J. Phaser Crystallographic Software. Journal Of Applied Crystallography 40 (4), 658–674, (2007).

(15) Vagin, A. A.; Steiner, R. A.; Lebedev, A. A.; Potterton, L.; McNicholas, S.; Long, F.; Murshudov, G. N. REFMAC5 Dictionary: Organization of Prior Chemical Knowledge and Guidelines for Its Use. Acta Crystallographica Section D: Biological Crystallography 60 (12 I), 2184–2195, (2004).

(16) Emsley, P.; Lohkamp, B.; Scott, W. G.; Cowtan, K. Features and Development of Coot. Acta Crystallographica Section D: Biological Crystallography 66 (4), 486–501, (2010).

(17) Winn, M. D.; Ballard, C. C.; Cowtan, K. D.; Dodson, E. J.; Emsley, P.; Evans, P. R.; Keegan, R. M.; Krissinel, E. B.; Leslie, A. G. W.; McCoy, A.; McNicholas, S. J.; Murshudov, G. N.; Pannu, N. S.; Potterton, E. A.; Powell, H. R.; Read, R. J.; Vagin, A.; Wilson, K. S. Overview of the CCP4 Suite and Current Developments. Acta Crystallographica Section D: Biological Crystallography 67 (4), 235–242, (2011).

(18) Afonine, P. V.; Grosse-Kunstleve, R. W.; Echols, N.; Headd, J. J.; Moriarty, N. W.; Mustyakimov, M.; Terwilliger, T. C.; Urzhumtsev, A.; Zwart, P. H.; Adams, P. D. Towards Automated Crystallographic Structure Refinement with Phenix.Refine. Acta Crystallographica Section D: Biological Crystallography 68 (4), 352–367, (2012).

(19) Chen, V. B.; Arendall, W. B.; Headd, J. J.; Keedy, D. A.; Immormino, R. M.; Kapral, G. J.; Murray, L. W.; Richardson, J. S.; Richardson, D. C. MolProbity: All-Atom Structure Validation for Macromolecular Crystallography. Acta Crystallographica Section D: Biological Crystallography 66 (1), 12–21, (2010).
